# CCN3/NOV promotes metastasis and tumor progression via GPNMB-induced EGFR activation in triple-negative breast cancer

**DOI:** 10.1038/s41419-023-05608-3

**Published:** 2023-02-03

**Authors:** Seogho Son, Hyungjoo Kim, Hogeun Lim, Joo-hyung Lee, Kyung-min Lee, Incheol Shin

**Affiliations:** 1 Department of Life Science, Hanyang University, Seoul, 04763 Korea; 2 Natural Science Institute, Hanyang University, Seoul, 04763 Korea; 3 Hanyang Institute of Bioscience and Biotechnology, Hanyang University, Seoul, 04763 Korea

**Keywords:** Breast cancer, Breast cancer

## Abstract

Triple-negative breast cancer (TNBC) is the most aggressive subtype of breast cancer. TNBC patients typically exhibit unfavorable outcomes due to its rapid growth and metastatic potential. Here, we found overexpression of CCN3 in TNBC patients. We identified that CCN3 knockdown diminished cancer stem cell formation, metastasis, and tumor growth in vitro and in vivo. Mechanistically, ablation of CCN3 reduced activity of the EGFR/MAPK pathway. Transcriptome profiling revealed that CCN3 induces glycoprotein nonmetastatic melanoma protein B (GPNMB) expression, which in turn activates the EGFR pathway. An interrogation of the TCGA dataset further supported the transcriptional regulation of GPNMB by CCN3. Finally, we showed that CCN3 activates Wnt signaling through a ligand-dependent or -independent mechanism, which increases microphthalmia-associated transcription factor (MITF) protein, a transcription factor inducing GPNMB expression. Together, our findings demonstrate the oncogenic role of CCN3 in TNBC, and we propose CCN3 as a putative therapeutic target for TNBC.

## Introduction

Breast cancer is one of the most frequently diagnosed cancers worldwide, and it has the highest rates of both diagnosis and mortality among women [1]. Breast cancer can be classified into molecular subtypes based on the presence of three receptors: estrogen receptor (ER), progesterone receptor (PR), and human epidermal growth factor receptor 2 (HER2). Triple-negative breast cancer (TNBC), a subtype that accounts for approximately 15% of invasive breast cancers, lacks expression of ER, PR, and HER2 [2]. TNBC is considered to be the most virulent subtype of breast cancer compared to others due to the rapid proliferation, metastatic potential and the lack of drug targets [3].

The CCN protein family, a type of matricellular protein, has been known to play a role in the interaction between extracellular matrix and cells [4]. The CCN family consists of six CCN proteins that share four conserved domains (except CCN5) and a signal peptide [4]. Each domain binds to proteins such as growth factors or receptors, which in turn facilitates the transmission of a signal into the cell [4]. Among these protein families, CCN3, also known as nephroblastoma overexpressed (NOV, NOVH), has been associated with cell migration, invasion, angiogenesis, adhesion, and proliferation in several cancer types like Ewing’s sarcoma, glioma, prostate cancer, hepatocellular carcinoma, clear cell renal cell carcinoma, chondrosarcoma, melanoma, intrahepatic cholangiocarcinoma and gastric cancer [5–18]. In addition, it has been reported that CCN3 induces actin cytoskeleton organization in breast cancer [19]. However, the role of CCN3 in TNBC remains elusive.

Epidermal growth factor receptor (EGFR) is a receptor tyrosine kinase that belongs to the EGFR family [20]. EGFR binds to various types of ligands such as epidermal growth factor (EGF), heparin-binding EGF-like growth factor (HB-EGF), and transforming growth factor alpha (TGFα) and is activated by forming homodimers or heterodimers with other receptors depending on the type of ligand [21]. Activated EGFR is involved in cell growth, differentiation, survival, and migration by activating signaling pathways such as the MAPK pathway through activation of the kinase domain inside the cell [20, 21]. Since abnormal activation of EGFR is closely related to the development of cancer, many studies have been conducted on EGFR in targeted therapy [20, 22]. Currently, various tyrosine kinase inhibitors or monoclonal antibodies that inhibit EGFR have been developed and used as therapeutic agents for cancers like non-small cell lung cancer and metastatic colorectal cancer [23, 24]. It has been confirmed that EGFR is also expressed in up to 90% of TNBC so that EGFR has been used as a target for TNBC treatment [25–27].

In this study, the molecular mechanism by which CCN3 acts on TNBC-specific tumorigenesis was explored. We confirmed that the expression of CCN3 was increased in a TNBC-specific manner and showed that the CCN3 was correlated with the basal-like phenotype and metastasis. CCN3 was found to induce cancer by interacting with the EGFR signaling pathway in TNBC. In addition, it was confirmed that glycoprotein nonmetastatic melanoma protein B (GPNMB) which shows increased expression due to CCN3, is involved in this regulation. Based on these results, we suggest a novel mechanism by which CCN3 acts on TNBC malignancy.

## Results

### CCN3 is overexpressed in TNBC patients and associated with poor clinical outcomes

**Fig. 1:**
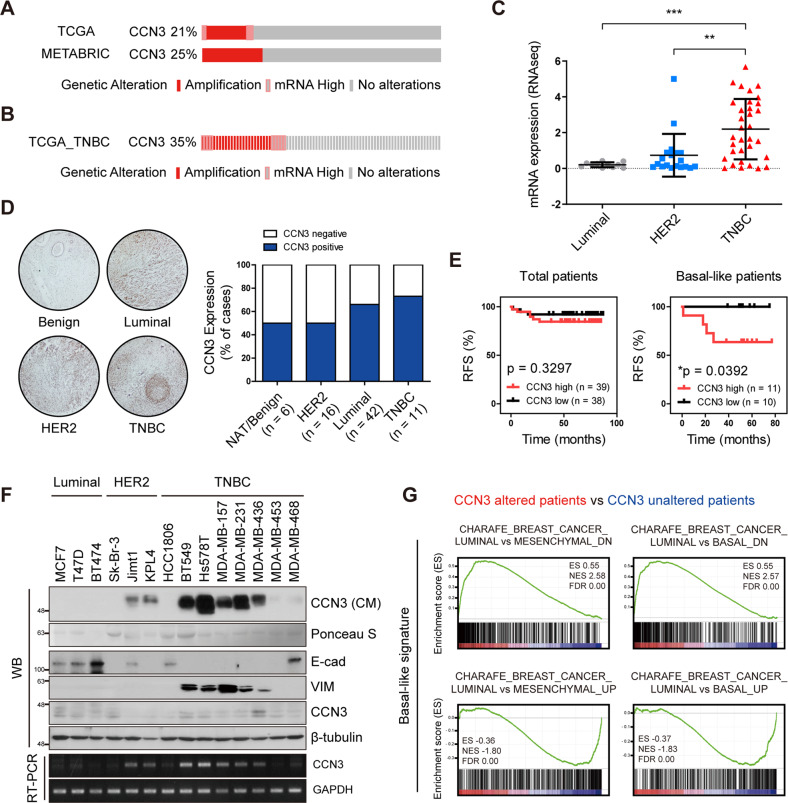
CCN3 highly expressed in TNBC and correlates with basal-like phenotype. **A** Oncoprint of breast cancer patients with gene amplification and mRNA up-regulation of CCN3 from TCGA (Cell, 2015) and METABRIC dataset. **B** Oncoprint of triple-negative breast cancer patients with gene amplification and mRNA up-regulation of CCN3 from TCGA (Cell, 2015) dataset. **C** CCN3 mRNA expression of breast cancer cell lines. mRNA expression data were obtained from CCLE dataset. Expression values were clustered by breast cancer molecular subtype (luminal, *n* = 11; HER2, *n* = 19; TNBC, *n* = 33). *P*-values were calculated with one-way ANOVA with a post-hoc Dunnett’s multiple comparison test (*p < 0.05, **p < 0.005). **D** Tissue array analysis of CCN3 expression with breast cancer patients. **E** Recurrence-free survival (RFS) plot of patients with CCN3 high versus low expression in GSE19615 dataset. *P*-values were calculated with log-rank test. **F** Western blot and RT-qPCR analysis of 14 breast cancer cell lines. Western blot analysis was assessed with whole cell lysate and precipitated protein from conditioned media. RT-qPCR analysis was assessed with cDNA of each cell line. β-tubulin was used as a loading control for whole cell lysate, and ponceau S staining intensity was used as a loading control for conditioned media. GAPDH was used as a loading control of RT-qPCR. **G** GSEA was performed with mRNA expression data from TNBC patients in the TCGA dataset (Cell, 2015). ES Enrichment score, NES Normalized enrichment score, FDR False discovery rate.

To identify the clinical implications of CCN3 in breast cancer, we checked the genetic alterations of CCN3 in breast cancer patients using a public database. The rate of *CCN3* alterations including copy number amplification and high mRNA expression accounted for 21 and 25% of entire breast cancer patients in TCGA and METABRIC, respectively (Fig. 1A). Notably, patients with *CCN3* alterations exhibited worse survival compared to *CCN3*-unaltered patients (Fig. S1A). More importantly, the rate of *CCN3* alterations was higher in TNBCs compared to non-TNBCs (35% vs. 21%; Fig. 1A, B). Using the Cancer Cell Line Encyclopedia (CCLE) database and public databases, we found that CCN3 expression is upregulated in TNBC (Fig. 1C and Fig. S1B). Furthermore, immunohistochemical analysis of tissue microarray showed that the CCN3 positive rate was highest in TNBC patients (Fig. 1D). In addition, in an analysis using a public database, overexpression of CCN3 was associated with poor clinical outcomes in patients with basal-like breast cancer. (Fig. 1E). We verified overexpression of CCN3 in the conditioned medium (CM) from TNBC cell lines with a panel of breast cancer cell lines (Fig. 1F and Fig. S1C). Since CCN3 has a signal peptide that leads to its extracellular localization [28], the majority of CCN3 protein was found in the CM, not in the cell lysates. (Fig. 1F). The mRNA expression pattern of *CCN3* among various breast cancer cell lines indicated by RT-PCR results in Fig. 1F coincides with CCN3 protein levels in the CM. Also, we found that *CCN3* mRNA expression was negatively correlated with *ESR1*, *PGR*, and *ERBB2* mRNA levels, respectively, in the CCLE dataset (Fig. S1D). Finally, gene set enrichment analysis (GSEA) showed that basal-like signatures are enriched in TNBC patients with *CCN3* alteration (Fig. 1G). Together, these data suggest that CCN3 is overexpressed in TNBC and associated with poor outcomes.

### CCN3 promotes EMT, migration, and invasion

**Fig. 2:**
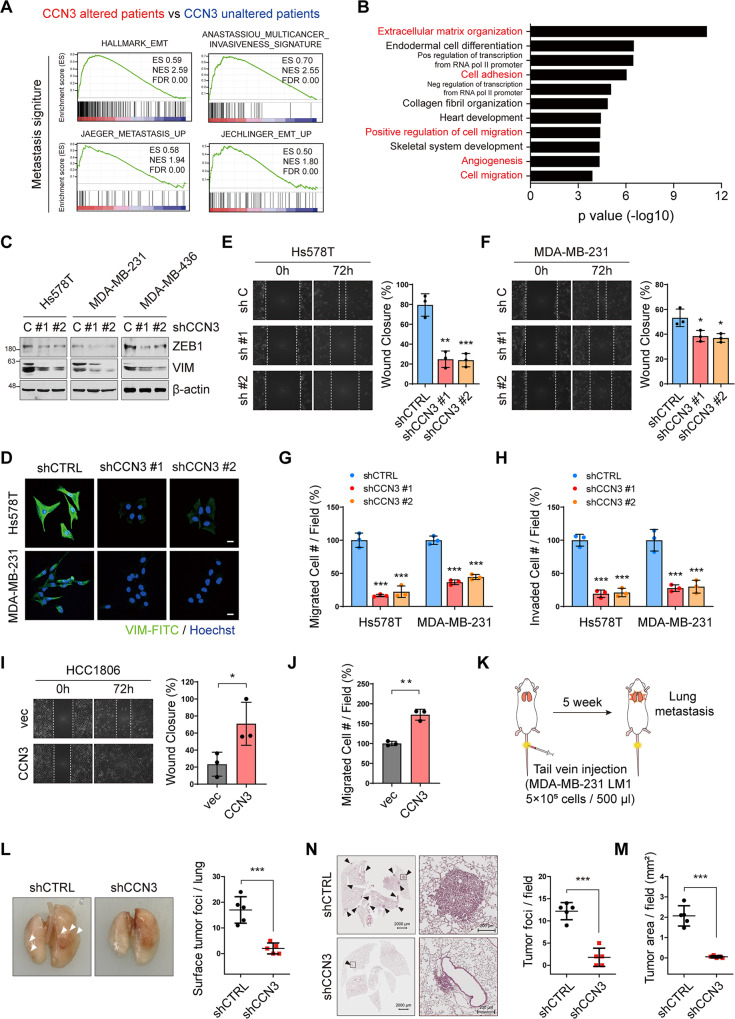
CCN3 promotes cell migration, invasion and EMT phenotype. **A** GSEA was performed with mRNA expression data from TNBC patients in TCGA (Cell, 2015) dataset. ES Enrichment score, NES Normalized enrichment score, FDR False discovery rate. **B** Gene ontology analysis of CCN3 altered TNBC patients versus unaltered TNBC patients of TCGA (Cell, 2015) dataset using DAVID (FDR < 0.05). **C**, **D** Western blot and immunofluorescence analysis with indicated antibody showed decreased expression of epithelial to mesenchymal transition marker proteins (scale bar = 20 μm). **E**, **F** Wound-healing assay of Hs578T and MDA-MB-231 CCN3 knockdown cell lines. The width of the wound area was monitored with a microscope at 24 h intervals. Mean ± SD (*n* = 3). *P*-values were calculated with two-way ANOVA with a Bonferroni posttest (*p < 0.05, **p < 0.005). **G**, **H** Transwell migration (**G**) and invasion (**H**) assays were assessed with Hs578T and MDA-MB-231 CCN3 knockdown cell. Each value was normalized with the value of the shCTRL. Mean ± SD (*n* = 3). *P*-values were calculated with a one-way ANOVA with a post-hoc Dunnett’s multiple comparison test (***p < 0.0005). **I** Wound-healing assay of HCC1806 CCN3 overexpression cell line. The width of the wound area was monitored with a microscope at 24 h intervals. Mean ± SD (*n* = 3). *P*-values were calculated with two-way ANOVA with a Bonferroni posttest (*p < 0.05, **p < 0.005). **J** Transwell migration assays were assessed for HCC1806 CCN3 overexpression cell lines. Each value was normalized with the value of the empty vector cell line. Mean ± SD (*n* = 3). P values were calculated using the two-tailed student *t*-test (n.s.; not significant, **p < 0.005). **K** Schematic experimental procedure for tail vein injection of tumor cells. MDA-MB-231 LM1 CCN3 knockdown cell line was used for injection. **L** Representative images showed tumor foci that has developed on the surface of the lung. Tumor foci on the lung surface were counted and quantified. Mean ± SD (*n* = 5). *P*-value was calculated with two-tailed student *t*-test (***p < 0.0005). **N** Representative images showed H&E staining of lung tissue. Black arrow indicated colonized tumor cells in each section (scale bar = 2000 μm, 200 μm). Tumor foci on each lung section were counted and quantified. Mean ± SD (*n* = 5). *P*-value was calculated with two-tailed student *t*-test (***p < 0.0005). **M** Colonized tumor areas on each lung section were measured and quantified using ImageJ software. Mean ± SD (*n* = 5). *P*-value was calculated with two-tailed student *t*-test (***p < 0.0005).

Interestingly, CCN3 expression was significantly correlated with vimentin in breast cancer cell lines, which is a mesenchymal marker that has been associated with epithelial to mesenchymal transition (EMT) and metastasis (Fig. 1F and Fig. S2A). Also, in the patient database, the expression of *CCN3* showed a significant positive association with EMT markers such as *VIM* and *ZEB1* (Fig. S2B). To gain more evidence for the correlation, we further interrogated the transcriptome data from TCGA. GSEA identified an enrichment of gene set signatures related to EMT and metastasis in *CCN3*-altered patients (Fig. 2A). Moreover, DAVID gene ontology (GO) analysis revealed that GO terms representing extracellular matrix organization, cell adhesion and cell migration, all of which are closely related to metastasis (Fig. 2B). Taken together, these data suggested that CCN3 may play a role in metastasis of TNBC.

To investigate the role of CCN3 in metastasis of TNBCs, we first established CCN3 knockdown cell lines using Hs578T, MDA-MB-231 and MDA-MB-436, all of which express high levels of CCN3. Then, we confirmed the downregulation of CCN3 in mRNA and protein levels (Fig. S2B, C). CCN3 knockdown in TNBC cell lines changed the mesenchymal-like morphology to a form with enhanced cell to cell junctions (Fig. S2D). Next, we examined the expression of the EMT marker gene in the CCN3 knockdown cell lines and found that ZEB1 and vimentin were downregulated by CCN3 knockdown (Fig. 2C, D). Furthermore, both cell migration and invasion were significantly attenuated by CCN3 knockdown (Fig. 2E–H and Fig. S2E, F). In addition, overexpression of CCN3 improved migration ability in HCC1806 cells that expressed low levels of CCN3 (Fig. 2I, J and Fig. S2G).

Next, we determined the role of CCN3 in metastasis in vivo. To do this, we employed an MDA-MB-231 LM1 model derived from lung lesions after metastasis [29]. We intravenously injected LM1 CCN3 knockdown or control cells into immunodeficient mice (Fig. 2K and Fig. S2H). We found that CCN3 knockdown suppresses the colonization of LM1 cells in the lungs, indicating the mitigated potential of metastasis (Fig. 2L, M). Together, these results suggest that CCN3 enhances migration and invasion of TNBC cells in vitro and in vivo.

### CCN3 enhances stem cell-like properties

**Fig. 3:**
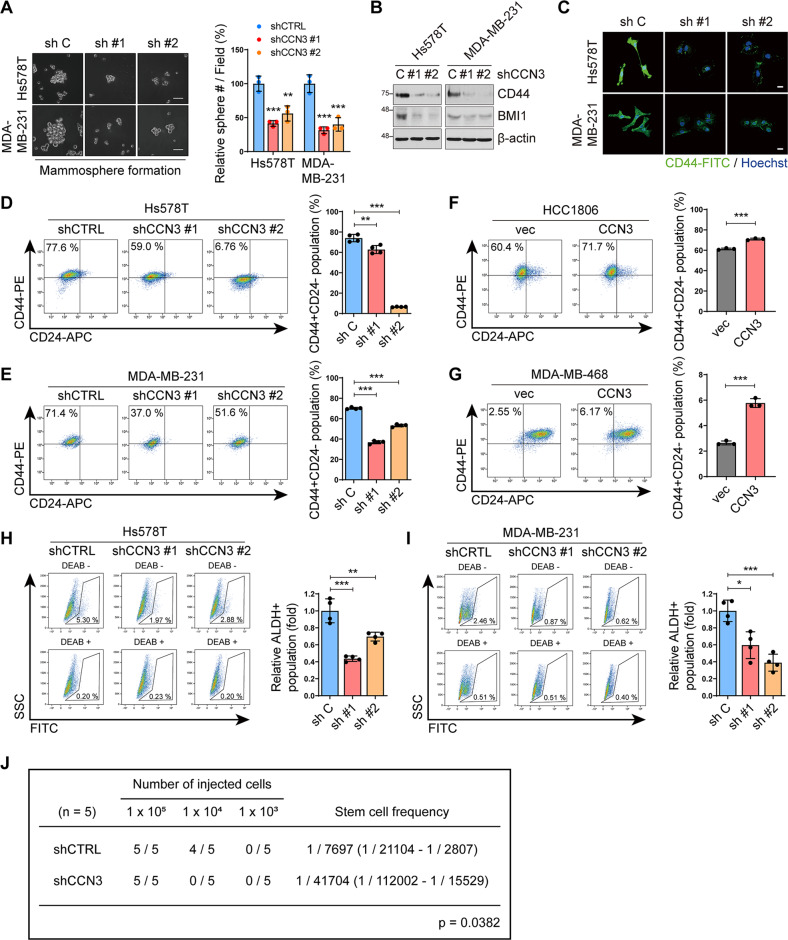
CCN3 enhances in vitro cancer stem cell-like phenotype. **A** Mammosphere formation assays were performed with Hs578T and MDA-MB-231 CCN3 knockdown cell lines (scale bar = 50 μm). Each value was normalized with the value of the shCTRL. Mean ± SD (*n* = 3). *P*-values were calculated with one-way ANOVA with a post-hoc Dunnett’s multiple comparison test (**p < 0.005, ***p < 0.0005). **B**, **C** Western blot (**B**) and immunofluorescence (**C**) analysis with indicated antibody showed decreased expression of cancer stem cell marker proteins (scale bar = 20 μm). **D**, **E** FACS analysis of CD44^+^ CD24^-^ cell population in the Hs578T (**D**) and MDA-MB-231 (**E**) CCN3 knockdown cell lines. Bar graphs indicated the percentage of CD44^+^ CD24^-^ cell population in each cell line. Mean ± SD (*n* = 4). *P*-values were calculated with one-way ANOVA with a post-hoc Dunnett’s multiple comparison test (***p < 0.0005). **F**, **G** FACS analysis of CD44^+^ CD24^-^ cell population in the HCC1806 (**F**) and MDA-MB-468 (**G**) CCN3 overexpression cell lines. Bar graphs indicated the percentage of CD44^+^ CD24^-^ cell population in each cell line. Mean ± SD (*n* = 3). *P*-values were calculated with a two-tailed student *t*-test (***p < 0.0005). **H**, **I** ALDEFLUOR assays were performed with Hs578T (**H**) and MDA-MB-231 (**I**) CCN3 knockdown cell lines. DEAB negative and positive plots indicated ALDH + cells and FITC negative respectively in each cell line. Bar graphs indicated the percentage of ALDH + cell population in each cell line. Mean ± SD (*n* = 4). *P*-values were calculated with one-way ANOVA with a post-hoc Dunnett’s multiple comparison test (**p < 0.005, ***p < 0.0005). **J** shCTRL and shCCN3 MDA-MB-231 LM1 cells were diluted and injected in the 4th mammary fat pad of NSG mice for limiting dilution assay.

We further investigated the effect of CCN3 on cancer stem cell (CSC) formation, which are known to be related to the EMT and metastatic ability of cancer cells [30, 31]. CCN3 ablation resulted in the reduction of sphere-forming ability, which is indicative of enrichment of CSC population (Fig. 3A). We observed that CCN3 knockdown reduced expression of CD44 and BMI1, both of which are known markers of CSC (Fig. 3B, C). In addition, flow cytometry analysis of CCN3 knockdown cell lines showed that the CD44^+^ /CD24^−^ population significantly decreased (Fig. 3D, E and Fig. S4A). Also, overexpression of CCN3 in HCC1806 and MDA-MB-468 cell lines increased the CD44^+^ /CD24^−^ population in flow cytometry analysis (Fig. 3F, G, Fig. S2H, J and Fig. S4B). Furthermore, we measured aldehyde dehydrogenase (ALDH) activity, which was another marker of CSC [32]. Aldefluor assay results show that ALDH activity significantly decreased in CCN3 knockdown cell lines (Fig. 3H, I). Finally, limiting dilution assay showed that knockdown of CCN3 significantly decreases CSC frequency in vivo (Fig. 3J). Altogether, these results suggest that CCN3 enhances the migration, invasion, and self-renewal ability of TNBC cells, all of which have been shown to promote metastasis.

### CCN3 promotes cell proliferation and enhanced clonogenic ability in TNBC cells

**Fig. 4:**
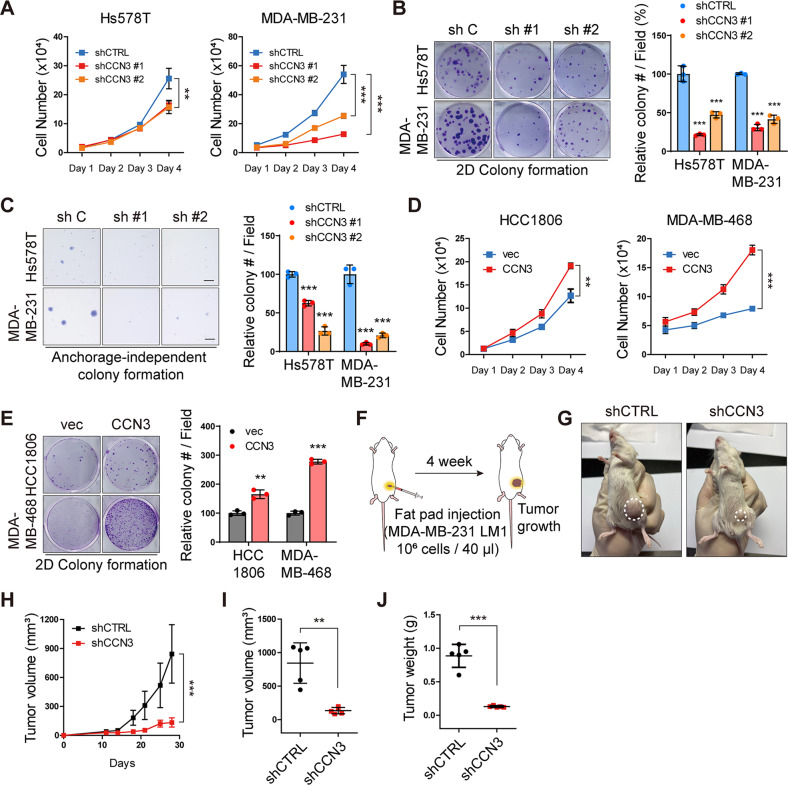
CCN3 promotes cell proliferation and enhanced clonogenic ability. **A** Cell proliferation assays were performed with Hs578T and MDA-MB-231 CCN3 knockdown cell lines. Mean ± SD (*n* = 3). *P*-values were calculated with two-way ANOVA with a Bonferroni posttest (**p < 0.005, ***p < 0.0005). **B** 2D colony formation assays were performed with Hs578T and MDA-MB-231 CCN3 knockdown cell lines. Each value was normalized with the value of shCTRL. Mean ± SD (*n* = 3). *P*-values were calculated with one-way ANOVA with a post-hoc Dunnett’s multiple comparison test (***p < 0.0005). **C** Anchorage-independent colony formation assays were performed with Hs578T and MDA-MB-231 CCN3 knockdown cell lines (scale bar = 50 μm). Each value was normalized based on the value of the shCTRL. Mean ± SD (*n* = 3). *P*-values were calculated with one-way ANOVA with a post-hoc Dunnett’s multiple comparison test (***p < 0.0005). **D** Cell proliferation assays were performed with HCC1806 and MDA-MB-468 CCN3 overexpression cell lines. Mean ± SD (*n* = 3). *P*-values were calculated with a two-way ANOVA with a Bonferroni posttest (n.s; not significant, **p < 0.005, ***p < 0.0005). **E** 2D colony formation assays were performed with HCC1806 and MDA-MB-468 CCN3 overexpression cell lines. Each value was normalized with the value of the empty vector cell line. Mean ± SD (*n* = 3). *P*-values were calculated with a two-tailed student *t*-test (n.s.; not significant, **p < 0.005, ***p < 0.0005). **F** Schematic experimental procedure for fat pad injection of tumor cells. MDA-MB-231 LM1 CCN3 knockdown cell line was used for injection. **G** Representative images showing xenograft tumor of LM1 CCN3 knockdown cell line 4 weeks after injection. **H** In vivo tumor growth curve of xenograft mouse. Mean ± SD (*n* = 5). *P*-values were calculated with two-way ANOVA with a Bonferroni posttest (***p < 0.0005). **I** Tumor volumes of xenograft mice at the last time point were quantified. Mean ± SD (*n* = 5). *P*-value was calculated with two-tailed student *t*-test (***p < 0.0005). **J** Tumor weights of each xenograft mouse were quantified. Mean ± SD (*n* = 5). *P*-value was calculated with two-tailed student *t*-test (***p < 0.0005).

CCN3 knockdown in Hs578T and MDA-MB-231 cells resulted in a decrease in cell proliferation, 2D colony-formation and anchorage-independent colony formation ability (Fig. 4A–C). In addition, we verified that monoclonal antibody neutralizing CCN3 mimics the anti-proliferative effect of CCN3 knockdown (Fig. S5). Similarly, overexpression of CCN3 significantly increased cell proliferation and 2D colony-formation compared to the control cell lines (Fig. 4D, E). To verify that these effects can be reproduced in a mouse model, we performed fat pad injection to assess the in vivo growth of a tumor (Fig. 4E). The results showed that the tumor growth was significantly reduced by CCN3 knockdown (Fig. 4F–J). In addition, IHC analysis validated that the expression of vimentin, CD44, and Ki67 were decreased in the tumors engrafted by CCN3 knockdown cells (Fig. S6). The reduction in proliferation by CCN3 knockdown was also confirmed in the lung metastasis model (Fig. S3). These results show that CCN3 also promotes growth of tumor cells in vitro and in vivo.

### CCN3 contributes to EGFR activation in triple-negative breast cancer

**Fig. 5:**
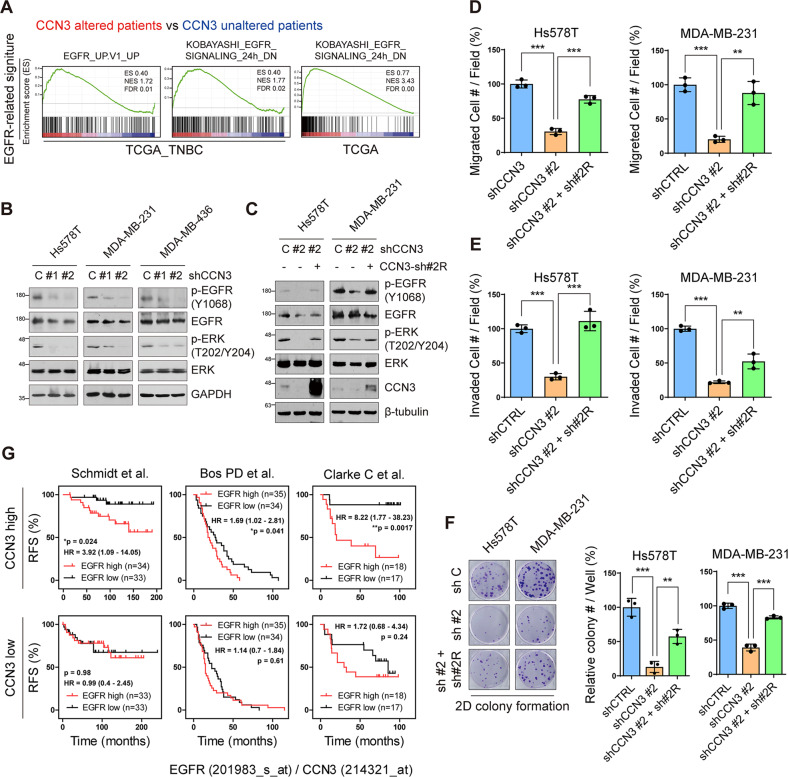
CCN3 correlates with EGFR signaling in TNBC. **A** GSEA was performed with mRNA expression data from TNBC and all patients in TCGA (Cell, 2015). Plots indicate EGFR-related gene signatures were enriched in CCN3 altered patients. ES; enrichment score, NES; normalized enrichment score, FDR; false discovery rate. **B** Western blot analysis of whole cell lysate of CCN3 knockdown cell lines with indicated antibody. GAPDH was used as a loading control. **C** Western blot analysis of whole cell lysate with indicated antibody. CCN3 knockdown cell line restored their expression by transfection with shRNA-resistant CCN3 construct. β-tubulin was used as a loading control. **D**–**E** Transwell migration (**D**) and invasion (**E**) assays were assessed with Hs578Ta and MDA-MB-231 CCN3 knockdown cell lines with or without shRNA resistant CCN3. Each value was normalized based on the value of the control cell line. Mean ± SD (*n* = 3). *P*-values were calculated with a one-way ANOVA with a post-hoc Tukey multiple comparison test (**p < 0.005, ***p < 0.0005). **F** 2D colony formation assays were performed with Hs578T and MDA-MB-231 CCN3 knockdown cell lines with or without shRNA resistant CCN3. Each value was normalized with the value of the control cell line. Mean ± SD (*n* = 3). *P*-values were calculated with one-way ANOVA with a post-hoc Tukey multiple comparison test (**p < 0.005, ***p < 0.0005). **G** Recurrence-free survival (RFS) plot of patients with EGFR high versus low expression in each dataset. Upper panel indicated patients filtered above median expression of CCN3 and bottom panel indicated patients filtered below median expression of CCN3. *P*-values were calculated with log-rank test.

To investigate the molecular mechanism of CCN3 on TNBC tumorigenesis, we examined changes in TNBC-specific signaling pathways in CCN3 altered patients. In GSEA using a TCGA database, EGFR signaling pathway-related gene signatures showed a significantly positive correlation with CCN3 altered patients (Fig. 5A). EGFR overexpression was reported to be observed in up to 90% of TNBC patients [25–27], and EGFR was expressed in most TNBC cell lines (Fig. S8). Based on these findings, we checked the activation of EGFR in the CCN3 knockdown cell lines by western blot analysis. The results showed that phosphorylation of EGFR and its downstream signaling protein ERK were significantly decreased by CCN3 knockdown in Hs578T, MDA-MB-231 and MDA-MB-436 cells (Fig. 5B). Ectopic expression of CCN3 using a shRNA resistant CCN3 construct rescued the suppressed EGFR/ERK phosphorylation in CCN3 knockdown cells (Fig. 5C). Furthermore, the forced expression of CCN3 restored migration, invasion, proliferation, and colony formation ability, all of which were mitigated in the CCN3 knockdown cells (Fig. 5D–F and Fig. S7). Finally, we found that higher expression of EGFR is significantly associated with poor clinical outcomes in CCN3-high TNBC patients, while it is not a prognostic factor in CCN3-low TNBC patients (Fig. 5G). Together, these results suggest that CCN3 overexpression is associated with EGFR activation in TNBC.

### CCN3 upregulates GPNMB expression in triple-negative breast cancer

**Fig. 6:**
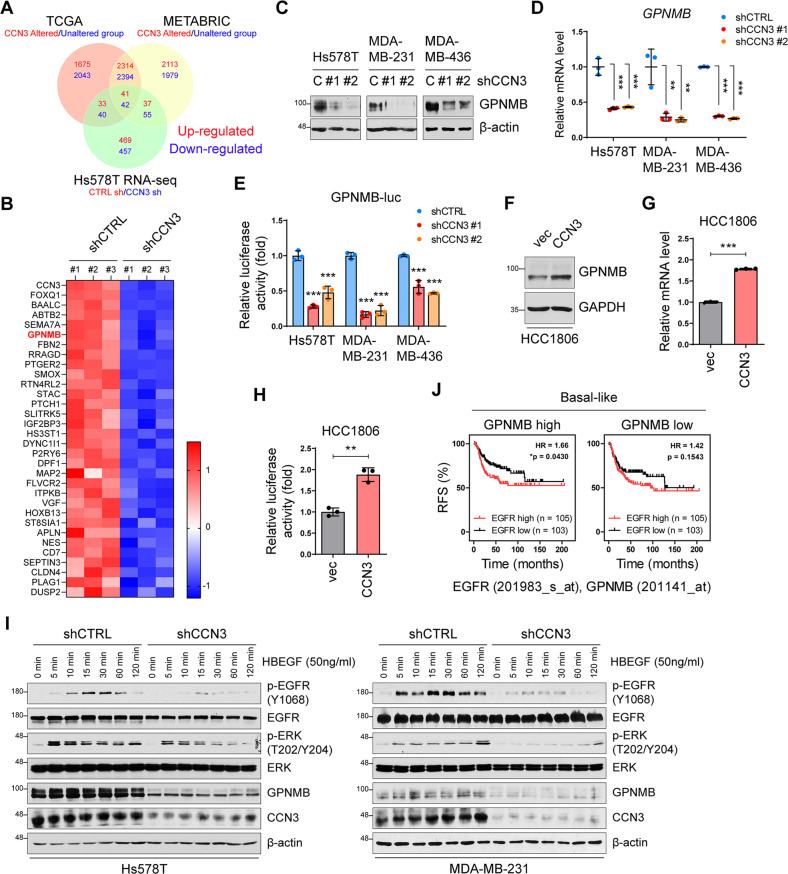
CCN3 regulates GPNMB expression. **A** The Venn diagrams represent the number of genes up- or down-regulated by CCN3 expression in each dataset. **B** The heatmap presents up-regulated genes by CCN3 from an RNA-seq dataset (< 2-fold, *p* < 0.0001). Up-regulated genes were common genes from Venn diagram (**A**). **C** Western blot analysis of whole cell lysate of CCN3 knockdown cell lines with indicated antibody. β-actin was used as a loading control. **D** RT-qPCR analysis showed the GPNMB mRNA level in Hs578T, MDA-MB-231 and MDA-MB-436 CCN3 knockdown cell lines. Each value was normalized with GAPDH. Mean ± SD (*n* = 3). *P*-values were calculated with one-way ANOVA with a post-hoc Dunnett’s multiple comparison test (**p < 0.005, ***p < 0.0005). **E** A dual luciferase assay was performed using pGL3-GPNMB-luc construct. pCMV-RL was used as an internal control. Mean ± SD (*n* = 3). *P*-values were calculated with one-way ANOVA with a post-hoc Dunnett’s multiple comparison test (***p < 0.0005). **F** Western blot analysis of CCN3 overexpression cell lines with indicated antibodies. GAPDH was used as a loading control. **G** RT-qPCR analysis showed GPNMB mRNA level in HCC1806 CCN3 overexpression cell lines. Each value was normalized with GAPDH. Mean ± SD (*n* = 3). *P*-values of CCN3 overexpression cell lines were calculated with two-tailed student *t*-test. (***p < 0.0005). **H** Dual luciferase assay was performed using pGL3-GPNMB-luc construct. pCMV-RL was used as an internal control. Mean ± SD (*n* = 3). *P*-values of CCN3 overexpression cell lines were calculated with two-tailed student *t*-test. (**p < 0.005). **I** Western blot analysis of whole cell lysate of CCN3 knockdown cell lines with indicated antibody. Each cell line treated with HBEGF (50 ng/ml) at the indicated time points. β-actin was used as a loading control. **J** Recurrence-free survival plot of patients with EGFR high versus low expression with basal-like breast cancer patients. Each panel indicated patients filtered by median expression of *GPNMB*. *P*-values were calculated with log-rank test.

To identify the molecular mechanism of CCN3-induced activation of EGFR, we performed RNA-sequencing with CCN3 knockdown Hs578T cells. We interrogated the transcriptome data to identify CCN3-target genes that potentially activate EGFR. In transcriptome analysis, we observed changes in EGFR or stem cell-related genes due to CCN3 knockdown (Fig. S9). To gain more comprehensive insight, we integrated it with transcriptome data from TCGA and METABRIC. We found that 83 genes out of 1174 genes that significantly up- or downregulated by CCN3 knockdown are associated with CCN3 alterations in clinical samples (Fig. 6A). Through this analysis, 41 genes commonly upregulated by CCN3 were identified. Then, 31 genes were obtained by applying a high cut-off (*p* < 0.0001) in RNA-seq results to the gene list showing a positive correlation with CCN3 expression (Fig. 6B). Among them, we found a gene called GPNMB. GPNMB, which is overexpressed in TNBC, has been previously shown to activate downstream signaling cascades by forming a heterodimer with EGFR, particularly when stimulated by HB-EGF [33–35]. We thus hypothesized that EGFR activation by CCN3 is mediated by GPNMB. We first confirmed that CCN3 knockdown resulted in a reduction of GPNMB expression at both the protein and mRNA level (Fig. 6C, D). To determine if CCN3 induces transcription of GPNMB, we generated a luciferase reporter that incorporates the promoter sequence of GPMNB. We found that GPNMB promoter activity was effectively decreased by CCN3 knockdown (Fig. 6E). Conversely, CCN3 overexpression caused an induction of GPNMB protein, mRNA, and transcriptional activity (Fig. 6F–H). Since CCN3 is a secreted protein, we also confirmed that GPNMB is increased by the addition of exogenous CCN3. Treatment with CM of shCTRL cell lines and recombinant human CCN3 (rhCCN3) could lead to the upregulation of GPNMB (Fig. S11A, B). In addition, CCN3 antibody co-treatment with abolished rhCCN3-induced GPNMB expression in HCC1806 (Fig. S11B, lane 3). Also, shRNA resistant CCN3 construct rescued the expression of GPNMB in CCN3 knockdown cell lines (Fig. S11C). It was also observed that the level of GPNMB was increased in a rhCCN3 treatment time-dependent manner (Fig. S11D).

We next determined whether CCN3 plays a critical role in the HB-EGF-dependent activation of EGFR. We verified that time-course treatment with purified recombinant human HB-EGF was able to activate the EGFR/ERK axis in Hs578T and MDA-MB-231 (Fig. 6I, lane 1 to lane 7). However, CCN3 knockdown diminished the prolonged activation of EGFR/ERK (Fig. 6I, lane 8 to lane 16). Finally, we found that the prognostic value of EGFR was limited in GPNBM-high TNBCs, not in GPNBM-low TNBCs (Fig. 6J). We also confirmed that levels of CCN3 mRNA were positively correlated with GPNMB mRNA expression in patient databases (Fig. S10A). Notably, the expression of *GPNMB* was upregulated in TNBC as was *CCN3* (Fig. S10B–C). Taken together, these results indicate that GPNMB mediates the CCN3-driven activation of EGFR in TNBCs.

### GPNMB induces migration, clonogenic ability and CSC-like properties

**Fig. 7:**
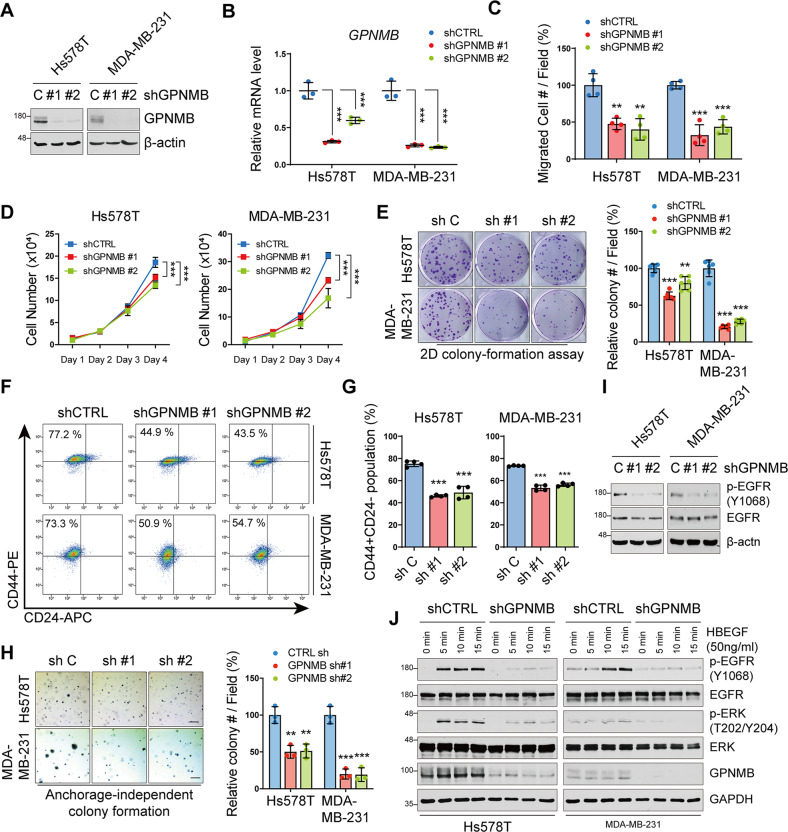
Knockdown of GPNMB drives anti-tumorigenic effect in vitro. **A** Western blot analysis of whole cell lysate of Hs578T and MDA-MB-231 GPNMB knockdown cell lines with indicated antibody. β-actin was used as a loading control. **B** RT-qPCR analysis showed GPNMB mRNA level in Hs578T and MDA-MB-231 GPNMB knockdown cell lines. Each value was normalized with GAPDH. Mean ± SD (*n* = 3). P values were calculated with one-way ANOVA with a post-hoc Dunnett’s multiple comparison test (***p < 0.0005). **C** Transwell migration assays were assessed with Hs578T and MDA-MB-231 GPNMB knockdown cell lines. Each value was normalized with the value of shCTRL. Mean ± SD (*n* = 4). *P*-values were calculated with one-way ANOVA with a post-hoc Dunnett’s multiple comparison test (***p < 0.0005). **D** Cell proliferation assays were performed with Hs578T and MDA-MB-231 GPNMB knockdown cell lines. Mean ± SD (*n* = 3). *P*-values were calculated with two-way ANOVA with a Bonferroni posttest (***p < 0.0005). **E** 2D colony formation assays were performed with Hs578T and MDA-MB-231 GPNMB knockdown cell lines. Each value was normalized with the value of CTRL cell line. Mean ± SD (*n* = 6). *P*-values were calculated with one-way ANOVA with a post-hoc Dunnett’s multiple comparison test (***p < 0.0005). **F**, **G** FACS analysis of CD44^+^CD24^-^ cell population in the Hs578T and MDA-MB-231 GPNMB knockdown cell lines. Bar graphs (**G**) indicated the percentage of CD44^+^CD24^-^ cell population in each cell line. Mean ± SD (*n* = 4). *P*-values were calculated with one-way ANOVA with a post-hoc Dunnett’s multiple comparison test (***p < 0.0005). **H** Anchorage-independent colony formation assays were performed with Hs578T and MDA-MB-231 CCN3 knockdown cell lines (scale bar = 50 μm). Each value was normalized based on the value of the CTRL cell line. Mean ± SD (*n* = 3). *P*-values were calculated with one-way ANOVA with a post-hoc Dunnett’s multiple comparison test (**p < 0.005, ***p < 0.0005). **I** Western blot analysis of whole cell lysate of GPNMB knockdown cell lines with indicated antibody. β-actin was used as a loading control. **J** Western blot analysis of whole cell lysate of GPNMB knockdown cell lines with indicated antibody. Each cell line treated with HBEGF (50 ng/ml) at the indicated time points. GAPDH was used as a loading control.

Since it was confirmed that the activity of EGFR can be enhanced by GPNMB, we further investigated whether CCN3-induced metastasis and tumor progression were mediated by GPNMB-induced mechanisms. GPNMB knockdown cell lines were constructed using two GPNMB-specific shRNA sequences, and knockdown was confirmed at the protein and mRNA levels (Fig. 7A, B). Cell migration, proliferation, and colony formation decreased in GPNMB knockdown cell lines (Fig. 7C–E). In addition, CSC population and anchorage-independent colony formation ability were significantly decreased (Fig. 7F–H and Fig. S12). As in the CCN3 knockdown cell line, EGFR activity decreased in the GPNMB knockdown cell lines, and reduced EGFR activity was also reproduced in the treatment with HB-EGF (Fig. 7I, J). These results show that GPNMB is a major factor in TNBC-specific tumorigenesis by CCN3.

### Expression of GPNMB by CCN3 is promoted by the Wnt/β-catenin/MITF axis

**Fig. 8:**
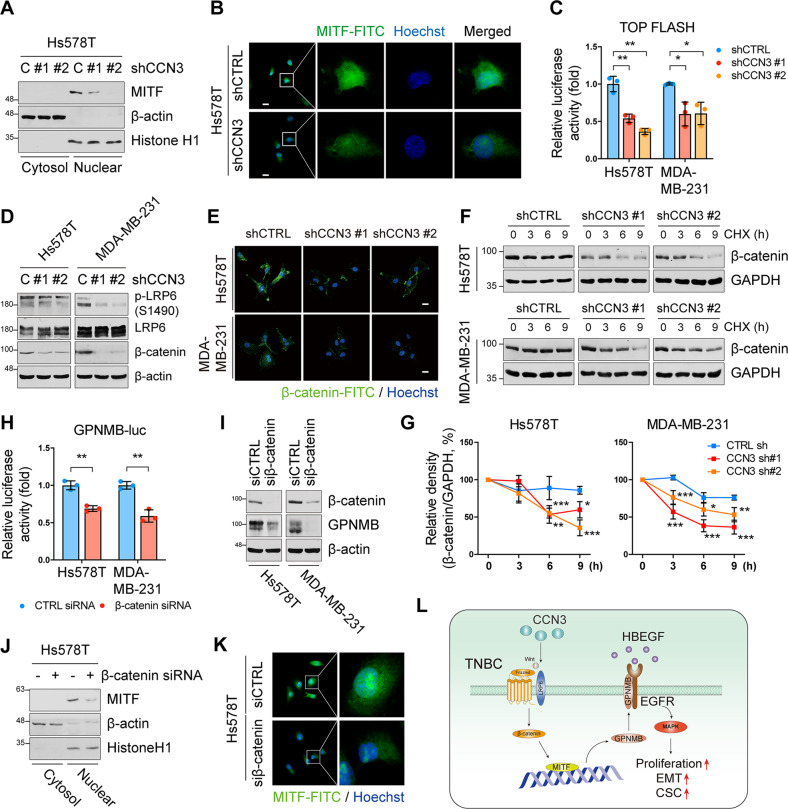
CCN3 regulates GPNMB expression by Wnt signaling induced MITF activation. **A** Western blot analysis of lysate isolated into cytoplasm and nuclear fraction of CCN3 knockdown cell line using the indicated antibodies. β-actin and histone H1 were used as a loading control for cytosol and nuclear fraction, respectively. **B** Immunofluorescence assay performed with indicated antibodies (scale bar = 20 μm). **C** Dual luciferase assay was performed using pSUPER-TOP FLASH construct. pCMV-RL was used as an internal control. Mean ± SD (*n* = 3). *P*-values were calculated with one-way ANOVA with a post-hoc Dunnett’s multiple comparison test (*p < 0.05, ***p < 0.0005). **D**, **E** Western blot (**D**) and immunofluorescence (**E**) analysis with indicated antibody showed decreased Wnt/β-catenin signaling proteins in Hs578T and MDA-MB-231 CCN3 knockdown cell lines (scale bar = 20 μm). **F**, **G** Cyclohexamide (CHX) chase assays were performed for measuring β-catenin degradation in Hs578T and MDA-MB-231 CCN3 knockdown cell lines. Each cell line treated with CHX (100 μg/ml) at the indicated time points. Quantification of western blot band was performed using ImageJ software. Mean ± SD (*n* = 3). *P*-values were calculated with two-way ANOVA with a Bonferroni posttest (*p < 0.05, **p < 0.005, ***p < 0.0005). **H** Western blot analysis of whole cell lysate with indicated antibody. Hs578T and MDA-MB-231 were treated with β-catenin siRNA for 48 h. β-actin was used as a loading control. **I** Dual luciferase assay was performed using a pGL3-GPNMB-luc construct. pCMV-RL was used as an internal control. Mean ± SD (*n* = 3). *P*-value was calculated using the two-tailed student *t*-test (**p < 0.005). **J** Western blot analysis of lysate isolated into cytoplasm and nuclear of β-catenin siRNA treated Hs578T cell line using the indicated antibodies. β-actin and histone H1 were used as a loading control for cytosol and nuclear fraction, respectively. **K** Immunofluorescence assay performed with indicated antibodies (scale bar = 20 μm). **L** Schematic description of CCN3 induced signaling cascade in TNBC.

Next, we sought a molecular pathway by which CCN3 induces GPNMB expression. Since GPNMB was regulated at the transcription level, we investigated a putative transcription factor for GPNMB, which can be regulated by CCN3. The previous study demonstrated that transcription factor microphthalmia-associated transcription factor (MITF) binds the promoter of GPNMB and regulates its expression [36]. TFEB and TFE3, other MiTF/TFE family members, are also known as upstream factors regulating GPNMB expression [37–39], and in our experimental results, knockdown of TFEB or TFE3 also reduces the expression of GPNMB phenocopying MITF knockdown (Fig. S13B–D). However, since only MITF was reduced by CCN3 knockdown in the cell lines tested, we hypothesized that CCN3 regulates GPNMB expression mainly via MITF (Fig. S13A). In addition, CCN3 knockdown resulted in a reduction of nuclear MITF protein (Fig. 8A, B). On the other hand, the level of MITF mRNA was not changed by CCN3 knockdown (data not shown). Therefore, we speculated that MITF is regulated by post-translational modification.

Because it was previously well documented that Wnt signaling can stabilize MITF protein [40], we first assessed the activity of Wnt signaling using pSUPER-TOPflash, a surrogate reporter of β-catenin-dependent transcriptional activation [41]. We found that CCN3 knockdown resulted in a reduction of the Wnt-dependent transcriptional activity (Fig. 8C). Moreover, levels of phospho- low-density lipoprotein receptor-related protein 6 (LRP6) and β-catenin, both of which belong to the canonical Wnt signaling cascade, were downregulated in CCN3-knocked down cells compared to control cells (Fig. 8D, E). Since the activation of Wnt signaling leads to stabilization of β-catenin protein by inhibiting proteasomal degradation [42], we assessed β-catenin protein stability. The cycloheximide (CHX) chase assay revealed that the stability of β-catenin protein was reduced by CCN3 knockdown (Fig. 8F, G).

Finally, we checked whether *GPNMB* transcription is promoted by the activation of Wnt signaling. Knockdown of β-catenin by siRNA resulted in a reduction in the nuclear fraction of MITF protein (Fig. 8J, K). Accordingly, the transcriptional activity and expression of GPNMB were downregulated by β-catenin knockdown (Fig. 8H, I and Fig. S14A). The activation of the EGFR/ERK pathway regulated by GPNMB was also reduced (Fig. S14B). Moreover, we confirmed that Wnt pathway activation by LiCl, an inhibitor of glycogen synthase kinase 3 (GSK3) that forms the β-catenin destruction complex, caused an increase in GPNMB expression at the mRNA and protein levels (Fig. S15). Collectively, these data imply that CCN3 enhances GPNMB transcription via the Wnt/β-catenin/MITF signaling axis, thereby activating EGFR signaling in TNBC (Fig. 8L).

## Discussion

In this study, we demonstrated the role of CCN3 in TNBC progression. We found that CCN3 is overexpressed in TNBC and associated with poor prognosis of breast cancer patients. In addition, we showed that CCN3 increases the growth, migration, invasion, clonogenic capacity and CSC formation of TNBC cells at both in vitro and in vivo. We found that these aggressive phenotypes are caused by the GPNMB-mediated EGFR activation. We verified that GPNMB was also causally associated with cell growth, migration, clonogenic ability, and CSC formation through EGFR activation. Finally, we suggested that CCN3 activates Wnt signaling, which in turn induces the expression of GPNMB.

CCN3 has been shown to act as a metastasis inducer by promoting the migration of cancer cells in glioblastoma, Ewing’s sarcoma, melanoma, chondrosarcoma, prostate cancer, clear cell renal cell carcinoma, and hepatocellular carcinoma [5, 7, 8, 10, 12, 13, 16, 43, 44]. Consistent with previous reports, our results also showed that CCN3 regulates breast cancer metastasis through the regulation of EMT and cell migration (Fig. 2). However, in contrast to role of CCN3 in metastasis, its impact on cell proliferation is somewhat controversial. In Ewing’s sarcoma, forced expression of CCN3 resulted in the suppression of cell proliferation [5]. Vallacchi and colleagues showed that the expression of CCN3 did not affect the tumor growth of melanoma [13]. On the other hand, CCN3 promotes tumor growth in hepatocellular carcinoma [9]. In glioblastoma cells, CCN3 increased cell proliferation via a platelet-derived growth factor receptor A (PDGFRα)-dependent mechanism [43]. Similarly, our results showed that proliferation was promoted by CCN3 in TNBC cell lines, but not in non-TNBC cells (Fig. 4 and data not shown). Based on these results, we speculate that CCN3 does not have a common regulatory function in all types of cancer but might show the diverse effect on different cancer cells depending on the gene expression pattern of each cancer cell type or different post translation modification of CCN3 in each cancer cells. Indeed, CCN3 is reported to differ in its glycosylation pattern depending on the cell types [15].

We confirmed that TNBC-specific upregulation of CCN3 among other breast cancer subtypes (Fig. 1C–D and Fig. S1B). The previous report also showed that the expression of ER has a negative correlation with CCN3 [45]. Interestingly, however, in this report, the expression level of CCN3 was decreased in tumor tissues compared to normal tissues. This finding may be contradictory to our results implying that CCN3 is involved in the malignancy of breast cancer. However, in the case of CCN3, the matricellular protein shows different glycosylation patterns depending on the tumor progression status, and the function varies according to the glycosylation pattern [11, 14]. Therefore, we speculated that the comparison of CCN3 expression in normal and tumor tissues requires further studies focusing on post-translational regulation rather than comparison of simple expression levels.

EGFR is overexpressed in up to 90% of TNBCs [25–27]. For this reason, many studies have been conducted to target EGFR in TNBC. Clinical studies testing EGFR-specific antibodies or tyrosine kinase inhibitors have been conducted, but no significant results were obtained [26, 46]. We speculated that these failures are related to the heterogeneity of TNBC, and that the EGFR signaling pathway might not act as a driver of cancer per se. According to a survival analysis using patient data, when CCN3 expression is high, the hazard ratio caused by EGFR expression is greatly increased (Fig. 5G). Based on these findings, we concluded that the expression of CCN3 can induce EGFR-dependent tumor progression as well as EGFR activation. In addition, it was confirmed that the cell viability was increased in the CCN3 knockdown cells compared to the control after treatment with EGFR inhibitor gefitinib (data not shown). These results suggest that CCN3 knockdown mitigates EGFR-dependent cell proliferation in TNBC cells. Thereby, we propose that the expression of CCN3 may be used as an index to check the potential of EGFR targeted therapy in TNBC.

GPNMB, also known as osteoactivin, promotes tumor growth, migration, invasion, and CSC formation in breast cancer [47–49]. GPNMB, which is overexpressed in TNBC, was suggested as a potential target of TNBC [33, 35]. GPNMB is also known to promote tumor initiation by increasing CSC formation and metastasis in TNBC [49]. Here, we showed that the CCN3 upregulates expression of GPNMB, which in turn contributes to activation of EGFR signaling. Consistent with CCN3, GPNMB knockdown attenuated tumorigenic phenotypes in vitro (Fig. 7). Although the oncogenic role of GPNMB has been suggested through preclinical studies including our current study, GPNMB-targeted therapies failed to elicit a durable response [50]. A recent study reported that inhibition of HSP90 increases the expression of GPNMB by inducing nuclear localization of MITF family, thereby increasing the sensitivity of GPNMB targeted therapy [51]. Consistently, we found that CCN3 increases GPNMB expression by upregulation of MITF and high expression of GPNMB is associated with poor outcome in only EGFR-high TNBCs (Figs. 8 and 6J). Taken together, we might suggest that CCN3 and EGFR are possible predictive biomarkers for use of GPNMB-targeted therapies.

Dysregulation of Wnt signaling has been associated with TNBC malignancy [52]. Herein, the interrogation of the TCGA dataset revealed a positive correlation between CCN3 expression and the Wnt/β-catenin pathway, which was assessed by GSEA (Fig. S9). In addition, we verified that the activation of Wnt signaling was attenuated by CCN3 knockdown in TNBC cells (Fig. 8C–F). Although we are not able to provide a solid mechanism(s) by which CCN3 activates Wnt signaling in the present study, previous studies suggest some putative mechanisms. First, CCN2 protein that belongs to the CCN protein family directly binds to LRP6, which in turn induces the activation of Wnt signaling [53]. Because CCN3 protein is structurally similar to CCN2 protein, we anticipate that CCN3 may interact with LRP6, consequently activating Wnt signaling. Second, our RNA-seq data revealed that the expression of various Wnt ligands was changed by CCN3 knockdown, which may imply a role of CCN3 in the regulation of Wnt ligands (Fig. S16B). Among them, we assumed that Wnt5A might be involved in CCN3-mediated Wnt/β-catenin signaling. We found that the expression of Wnt5A was significantly reduced by CCN3 knockdown (Fig. S16C). Based on these findings, we speculate that CCN3 could directly or indirectly regulate Wnt signaling, and further detailed experimental evidence are needed to fully support our hypothesis.

In summary, we identified that CCN3 is amplified and plays a crucial role in proliferation, metastasis, and CSC enrichment in TNBC via GPNMB induced EGFR activation. Therefore, we propose CCN3 as both a prognostic biomarker and a putative therapeutic target of TNBC.

## Materials and methods

### Bioinformatic analysis of public database

*CCN3* copy number alteration and mRNA expression data of breast cancer patients were analyzed using TCGA [54] and METABRIC [55, 56] databases obtained from cBioPortal (http://cbioportal.org/) [57, 58]. In each database, patients were classified as *CCN3* altered patients if their *CCN3* copy number was amplified or they showed high *CCN3* mRNA expression, and each analysis was performed using the classified patient group. GSE20194 and GSE25066 were used for *CCN3* and *GPNMB* expression data for each breast cancer subtype. An overall survival analysis of CCN3 altered/unaltered patients was performed using cBioPortal. Gene expression correlation analysis of breast cancer patients was performed in cBioPortal using mRNA expression (log RNA Seq V2 RSEM) of TCGA database. Gene ontology analysis was performed using the Database for Annotation, Visualization and Integrated Discovery (DAVID) (https://david.ncifcrf.gov/) [59]. Gene set enrichment analysis (GSEA) was performed using mRNA expression data from the TCGA database. The gene set used for GSEA was obtained from the Molecular Signature Database (MSigDB) and published gene signatures [60]. Gene expression data for breast cancer cell lines were obtained from RNA sequencing data of the cancer cell line encyclopedia (CCLE) database and were analyzed using the DepMap portal (https://depmap.org/portal/). GSE11121, GSE12276, GSE19615, GSE42568 and integrated cohorts in the Kaplan-Meier plotter (http://kmplot.com/analysis/) were used for breast cancer patient survival analyses. Each analysis performed in the Kaplan-Meier plotter using a designated probe of genes that compared patients in the upper and lower terciles of expression for each gene [61].

### Cell culture

Hs578T, MDA-MB-231, MDA-MB-436, MDA-MB-468, and HCC1806 human breast cancer cell lines were obtained from American Type Culture Collection (ATCC, Manassas, VA, USA). MDA-MB-231 LM1 was kindly provided by Professor SJ Lee (Hanyang University, Seoul, Korea). Hs578T, MDA-MB-231, MDA-MB-436, MDA-MB-468, and MDA-MB-231 LM1 were maintained in DMEM (Corning, NY, USA) containing 10% fetal bovine serum (Corning, NY, USA) and 1% antibiotic-antimycotic (Gibco; Thermo Fisher Scientific, Inc., Waltham, MA, USA). HCC1806 was maintained in RPMI 1640 (Corning, NY, USA) containing 10% fetal bovine serum (Corning, NY, USA) and 1% antibiotic-antimycotic (Gibco; Thermo Fisher Scientific, Inc., Waltham, MA, USA) All cell lines were incubated at 37 °C in 5% CO_2_ in a humidified atmosphere.

### Antibodies and reagents

All antibodies used in this study and their applications are listed in Table S1. Hematoxylin (HHS32), eosin (318906) and polybrene (TR-1003) were purchased from Sigma-Aldrich (St. Louis, MO, USA). BCA Protein Assay Kit (23225) and Hoechst 33342 (H1399) were purchased from Thermo Fisher Scientific (Waltham, MA, USA). Lipofectamine 3000 transfection reagent (L3000015) was purchased from Invitrogen (Carlsbad, CA, USA). Recombinant human CCN3 protein (1640-NV) was purchased from R&D systems (Minneapolis, MN, USA).

### Plasmids and siRNAs

CTRL and CCN3/GPNMB shRNA vectors were generated by insertion of each shRNA sequence in the pSUPER-retro-puro viral vector (OligoEngine, Seattle, WA, USA) according to the manufacturer’s protocol. Sequences of each shRNA are listed in Table S2. For the generation of a CCN3 expression vector, the coding sequence of CCN3 was amplified by PCR. Then, PCR products were cloned into pcDNA 3.1-myc-his-A vector using a HindIII/XhoI restriction site. To generate an expression vector for CCN3 stable overexpression cell lines, PCR amplification was performed using pcDNA 3.1-CCN3-myc-his as a template, and this product was cloned into pBMN-I-EGFP vector using a BamHI/EcoRI restriction site. For the generation of shRNA#2 resistant CCN3 expression vectors, CCN3 construct harboring wobble mutations were amplified using a two-step PCR procedure employing an overlap extension PCR method as described previously [62]. The PCR products were cloned into a pcDNA 3.1-myc-his-A vector using a HindIII /XhoI restriction site. To construct an expression vector for purification of recombinant human HB-EGF, the coding sequence of active HB-EGF was obtained through PCR amplification. The products were cloned into a pET-16b vector using an NdeI /BamHI restriction site. The GPNMB promoter region was obtained through PCR amplification using Hs578T genomic DNA as a template. The sequence of the GPNMB promoter was determined as the upper 1 kb region of the first exon of GPNMB, and this sequence was referred to S. Loftus et al. 2009 [36]. The PCR product was cloned into a pGL3-based vector using XhoI/HindIII restriction sites, which was named GPNMB-luc. All primers used for plasmid construction are listed in Table S3, and all plasmids were confirmed through sequencing. Control siRNA (sc-37007) β-catenin siRNA (sc-29209), MITF siRNA (sc-35934), TFEB siRNA (sc-38509), TFE3 siRNA (sc-38507) were purchased from Santa Cruz Biotechnology. Plasmid and siRNA were transfected using Lipofectamine 3000 according to the manufacturer’s protocol.

### Generation of stable cell lines

For generation of knockdown cell lines, 293 T cells were seeded at a density of 1 × 10^6^ cells on 100 mm dishes. After 24 h, cells were transfected with pSUPER-CTRL shRNA or pSUPER-CCN3/GPNMB shRNAs along with pCMV-VSVG and pCMV-Gag-Pol (8:3:4 mass ratio). After 48 h, retroviral particles were collected from the culture medium of 293 T cells. Hs578T, MDA-MB-231, MDA-MB-436 and MDA-MB-231 LM1 cells were treated with media containing retroviral particles and 10 μg/mL of polybrene (Sigma, St. Louis, MO, USA). CTRL and CCN3/GPNMB knockdown clones were selected by treating cells with puromycin. The selected clones were subjected to western blot and RT-qPCR to confirm their decreased expression of target genes. For generation of overexpression cell lines, 293 T cells were seeded at a density of 1 × 10^6^ cells on 100 mm dishes. After 24 h, cells were transfected with pBMN-EGFP-vec or pBMN-EGFP-CCN3 along with pCMV-VSVG and pCMV-Gag-Pol (8:3:4 mass ratio). After 48 h, retroviral particles were collected from the culture medium of 293 T cells. HCC1806 cells were treated with media containing retroviral particles and 10 μg/mL of polybrene (Sigma, St. Louis, MO, USA). Cell lines were constructed by sorting only the cells expressing EGFP using flow cytometry.

### Immunohistochemistry (IHC)

For IHC of breast cancer patient tissue arrays, tissue array slides were purchased from US Biomax (BR1503f, Rockville, MD, USA). The tissue slide contains 3 cases of adjacent normal breast tissue (NAT) and breast fibroadenoma, 2 cases of breast cystosarcoma phyllodes, 7 cases of breast intraductal carcinoma, and 60 cases of breast invasive ductal carcinoma. Each case has duplicate cores. Paraffin-embedded tissue slides were incubated in a dry oven at 60 °C for 1 h. The slide was deparaffinized in xylene and rehydrated by sequential incubation in 100%, 95%, 80%, and 70% ethanol for 5 min each. After hydration, heat-induced antigen retrieval was conducted using sodium citrate buffer (10 mM sodium citrate, 0.05% Tween 20, pH 6.0). Then, the slide was incubated overnight at 4 °C in a humidified chamber with the primary antibody diluted in PBS with 3% horse serum. After washing three times with PBS, the slide was incubated with biotin-conjugated secondary antibody for 1 h. The avidin–biotin complex reaction was generated according to the manufacturer’s protocol (PK-6101; PK-6102, Vector Laboratories, Burlingame, CA, USA). The color reaction was performed using DAB (Vector Laboratories), and counter staining was performed using hematoxylin.

For IHC of xenograft tumor tissue sections, tumor tissues were isolated and fixed in 10% formalin overnight. After embedding the tissue in paraffin, it was sectioned to a thickness of 5 μm using a microtome and was prepared by mounting it on a silane-coated slide. The following procedure is the same as described above.

### Cell proliferation assay

Cells were seeded at 1 × 10^4^ cells per well in 12well culture plates. Cells were harvested and counted with a hemocytometer every 24 h for 4 days.

### Dual luciferase assay

Cells were seeded at 2 × 10^4^ cells per well in 24-well culture plates. After 24 h, cells were transfected with reporter constructs (500 ng) and pCMV-RL (2.5 ng) as an internal control using Lipofectamine 3000 (Invitrogen). Dual luciferase assays were performed using a Dual-Luciferase Reporter Assay System (Promega, San Luis Obispo, CA, USA) according to the manufacturer’s protocol 48 h after transfection.

### Immunofluorescence

Cells were seeded at 2 × 10^4^ cells per well in 12-well culture plates with a coverslip. After 24 h, cells were washed with PBS and fixed for 10 min using a 3.7% formaldehyde solution. After that, 0.5% Triton X-100 was used for permeabilization, and blocking was performed for 30 min using 3% or skim milk. For staining of target proteins, the primary antibody and the fluorescence-conjugated secondary antibody were diluted in 1% skim milk and incubated with the cells for 1 h each. For staining of DNA, Hoechst 33342 dye (1 μg/ml) was incubated with the cells for 10 min after target protein staining. Immunofluorescence was confirmed using a Nikon C2 Si-plus confocal microscope (Nikon, Tokyo, Japan) and data analysis was performed using ImageJ software.

### Subcellular fractionation

The protocol S. Kim et al. 2021 [63] was used with slight modifications. Cells were washed once with PBS and lysed with cytoplasmic extraction buffer (10 mM Tris at pH 7.4, 10 mM KCl, 3 mM MgCl2, 0.5% NP-40, 1 mM PMSF). After 20 min on ice, cells were centrifuged at 13,000 rpm, 4c, 5 min to obtain a supernatant. Supernatants were transferred into a new 1.5 ml tube (cytoplasmic fraction). The pellets were washed with 500 μL nuclear washing buffer (10 mM HEPES at pH 7.9, 10 mM KCl, 1.5 mM MgCl2, 0.34 M sucrose) and centrifuged at 13,000 rpm at 4 °C for 5 min two times. After discarding supernatant, pellets were homogenized with a 29 G syringe in a nuclear extraction buffer (20 mM Tris at pH 8.0, 0.42 M NaCl, 0.2 mM EDTA, 10% glycerol, 2 mM DTT, 1 mM PMSF). After 20 min on ice, pellets were centrifuged at 16,000 rpm, 4c, 20 min to obtain a supernatant. Supernatants were transferred into a new 1.5 ml tube (nuclear fraction). Each fraction was used for further experiments.

### Conditioned media

For conditioned media used for western blot analysis, cells were seeded in 100 mm culture dishes and incubated until reaching approximately 90% confluency. After this, they were washed with PBS and changed with serum-free media. After 24 h, the whole medium was harvested and centrifuged at 1000 g to remove cells and debris. Proteins were precipitated from the supernatant using the methanol/chloroform method and were used for western blot analysis. For conditioned media used to treat the cells, cells were seeded in T75 culture flasks and incubated until reaching approximately 90% confluency. After which they were washed with PBS and changed with fresh complete media. After 24 h, the whole medium was harvested and centrifuged at 1000 g to remove cells and debris. The supernatant was filtered using a 0.45 um syringe filter, and the 1/4 dilution was mixed with fresh media and used for further experiments.

### Quantitative real-time polymerase chain reaction (qRT-PCR)

Total cell RNA was isolated with TRIzol reagent (Molecular Research Center, Cincinnati, OH, USA). Subsequently, RT-PCR was performed using a TOYOBO RT-PCR kit (TOYOBO, Osaka, Japan). Quantitative real-time PCR was performed with a SYBR FAST qPCR kit (Kapa Biosystems) in a Thermal Cycler Dice (Takara, Otsu, Shiga, Japan) according to the manufacturer’s protocols. C(t) values were obtained through the crossing point (CP) method and normalized using GAPDH. The primers used in the experiment were as follows: CCN3 forward, 5’- TCG CAG CGC GGA CCC CAG CAA -3’ and reverse, 5’- CCA GCT GAC AGC GGG GCA CAC -3’; GPNMB forward, 5’- TCA CCC AGA ACA CAG TCT GC -3’ and reverse, 5’- CAG ACC CAT TGA AGG TTC GT -3’; MITF forward, 5’- CAT GCA ACA GAG AGT GCC -3’ and reverse, 5’- GTA CTG CTT TAC CTG CTG CC -3’; TFEB forward, 5’- CTG GAC GAT GTC CTT GGC TA -3’ and reverse, 5’- TGT GAT TGT CTT TCT TCT GCC G -3’; TFE3 forward, 5’- TGT GTA CAG TAG TCA AGG CGT -3’ and reverse, 5’- AGT GCC CAG TTC CTT GAT CC -3’; WNT5A forward, 5’- TAC GAG AGT GCT CGC ATC CTC A -3’ and reverse, 5’- TGT CTT CAG GCT ACA TGA GCC G -3’; GAPDH forward, 5’- GGC AAA TTC CAT GGC ACC GTC -3’ and reverse, 5’- GCC AGC ATC GCC CCA CTT GAT -3’.

### Transwell migration and invasion assay

Cell migration and invasion assays were performed using 8 μm pore size transwell chambers (Corning, NY, USA). The lower chamber was filled with complete culture medium (DMEM with 10% FBS). Cells were suspended in DMEM with 1% FBS and 1 × 10^4^ cells were seeded in the upper chamber. After 24 h, transwell chambers were washed with PBS and stained with 0.5% crystal violet. The number of cells on the bottom surface of the polycarbonate membrane was counted using an optical microscope. For cell invasion assays, the upper chamber was coated with Matrigel (Corning, NY, USA) 2 h prior to cell seeding.

### Wound healing assay

Cells were seeded in 6-well culture plates and incubated until reaching approximately 100% confluency. After this, they were washed with PBS and changed with serum-free media. Wounded areas were generated by scraping the plate monolayer with a pipette tip, and cells were treated with cycloheximide (10 μM) to inhibit protein synthesis. Wound closure was measured under a microscope at 24 h intervals. The wound area at each time point was normalized using the average of the initial wound area.

### Two-dimensional colony formation assay

Cells were seeded at 200 cells per well in 6-well culture plates and incubated for 7 to 10 days at 37 °C in a CO_2_ incubator. After incubation, the cells were washed with PBS and stained with 0.5% crystal violet for 30 min. The cells were then washed twice with PBS and observed with an optical microscope. Colonies were counted and quantified by ImageJ software.

### Anchorage independent colony formation assay

For the Anchorage independent colony formation assay, 6 well plates were coated with a mixture of 1.8% agarose (Duchefa Biochemie) and 2X complete DMEM (1:1 volume ratio). After 3 h in a 37 °C incubator for hardening, cells were seeded at 1.5 × 10^3^ in each well mixed with 0.9% agarose (1:1 volume ratio). Wells were covered with 1 ml media and incubated for 10–14 days. After incubation, the cells were washed with PBS and stained with 0.05% crystal violet for 2 h. The cells were then washed with PBS for several days and were observed with an optical microscope. Colonies were counted and quantified by ImageJ software.

### Flow cytometry

For analysis of the CD44^+^CD24^-^ cancer stem cell population, cells were seeded at 1 × 10^6^ cells in 100 mm culture plates. Cells were washed once with PBS and stained with fluorescence-conjugated antibodies (CD44-PE; BioLegend, CD24- Alexa Fluor 647; Santa Cruz Biotechnology) for 30 min. Flow cytometry was performed with a BD FACS Canto II flow cytometer (BD Biosciences, Franklin Lakes, NJ, USA), and data analysis was performed using FlowJo software. To quantify aldehyde dehydrogenase (ALDH) activity, an ALDEFLOUR assay (StemCell Technologies) was performed according to the manufacturer’s protocol.

### Western blots

Cells were washed once with PBS and lysed with lysis buffer (20 mM Tris-HCl, pH 7.4, 0.1 mM EDTA, 150 mM NaCl, 1% NP-40, 0.1% Triton X-100, 0.1% SDS, 20 mM NaF, 1 mM Na_3_VO_4_, 1× protease inhibitor cocktail, Roche, Basel, Switzerland). Equal amounts of protein in cell lysates were determined by standardization with the BCA Protein Assay Kit (Thermo Fisher Scientific). Proteins were resolved by SDS-PAGE and transferred to nitrocellulose membranes (Whatman GmbH, Dassel, Germany). After blocking with 5% skim milk in TBST at RT for 1 h, membranes were incubated with primary antibodies overnight at 4 °C, followed by 2 h incubation with HRP-conjugated secondary antibodies at RT. Protein bands were visualized with Dyne ECL (Dyne, Seongnam, Korea).

### Mammosphere formation assay

Cells were seeded at 6 × 10^2^ cells per well in poly-HEMA coated 12-well culture plates. Cells were maintained in sphere-forming medium (DMEM/F12 containing 10 ng/ml bFGF, 20 ng/ml EGF (Peprotech, Rocky Hill, NJ, USA) and 1% B27 supplement (Life Technologies, Carlsbad, CA, USA). After 7 to 10 days, spheres were observed and counted with an optical microscope.

### Animal experiments

NSG mice 8 to 10 weeks old (Orient Bio, Seoul, Korea) were used in all experiments. For intravenous injection, 5 × 10^5^ MDA-MB-231 LM1 shCTRL /shCCN3 cells were suspended in 500 μl PBS and injected through the lateral tail vein of the mice. Five weeks after injection, the mice were sacrificed, and the lungs were isolated. Isolated lungs were fixed in 3.7% paraformaldehyde, and tumor foci that occurred on the surface of the lung were measured by the naked eye with a magnifier. The tissue was then embedded in paraffin for hematoxylin and eosin tissue staining. For fat pad injection, 1 × 10^6^ MDA-MB-231 LM1 shCTRL /shCCN3 cells were suspended in 40 μl PBS and injected through the 4th mammary fat pad of the mice. After injection, the tumor volume was measured using calipers at intervals of twice a week. The tumor volume was calculated using the following formula: (longest diameter × perpendicular diameters^2^) / 2. Mice were sacrificed 4 weeks after injection and primary tumors were isolated. Tumor tissues were weighed and fixed in 3.7% paraformaldehyde for histologic analyses. For limiting dilution assay, serially diluted MDA-MB-231 LM1 shCTRL /shCCN3 cells (1 × 10^5^, 1 × 10^4^, 1 × 10^3^) were suspended in 40 μl PBS and injected through the 4th mammary fat pad of the mice. Mice were observed for tumor formation for 4 weeks. Extreme limiting dilution analysis (ELDA) was used to estimate the frequency of tumor-initiating cells [64].

### RNA sequencing

For RNA sequencing analysis, the total RNA of Hs578T shCTRL /shCCN3 #2 was isolated using TRIzol reagent (Molecular Research Center, Cincinnati, OH, USA). Total RNA integrity was checked using an Agilent Technologies 2100 Bioanalyzer with an RNA Integrity Number (RIN) value greater than or equal to 7. The library was prepared using the TruSeq Stranded mRNA LT Sample Prep Kit (Illumina, San Diego, CA, USA) according to the manufacturer’s protocols. The constructed libraries were subjected to NovaSeq 6000 systems for sequencing on the Macrogen. All procedures were conducted following the manufacturer’s instructions.

### Cycloheximide chasing assay

Cells were seeded at a density of 1 × 10^6^ cells per 100 mm dish. After 24 h, cells were treated with 100 μg/ml of cycloheximide (Sigma-Aldrich, St. Louis, MO, USA) to block protein synthesis. After each time point, cells were harvested, and the changed protein was analyzed by western blot.

### Purification of recombinant human HB-EGF

E. coli Rosetta (DE3) cells were transformed with pET-16b containing active HB-EGF sequence and incubated in 5 ml of LB with ampicillin for 16 h at 37 °C. The whole medium was inoculated into 1 L of fresh medium and incubated until OD600 reached 0.3–0.4. IPTG was added at 1 mM and further incubated at 37 °C for 2 h. Cells were harvested and resuspend with PBS. Then, whole cells were lysed with sonication. His-tagged HB-EGF proteins contained in the supernatant were incubated with Ni Sepharose high performance (GE healthcare, Chicago, Illinois, USA) for 2 h at 4 °C. Ni beads were washed twice with wash buffer (PBS containing imidazole 20 mM, NaCl 500 mM) and eluted with elution buffer (PBS containing imidazole 500 mM, NaCl 500 mM). Purified proteins were desalted and exchanged with PBS buffer using Micro Bio-Spin P-6 columns (Bio-Rad, Hercules, CA, USA). Protein concentration was measured based on A280 value and was confirmed by SDS-PAGE with BSA.

### Statistics

All experiments were performed with at least three independent trials. Data are expressed as means ± standard deviations. All statistical analyses were conducted using GraphPad Prism version 5.03 for Windows (GraphPad Software, San Diego, CA USA). The statistical significance between the two groups was calculated with the two-tailed student *t*-test. For multiple groups of 3 or more, a one-way ANOVA (post hoc Dunnett’s test or Tukey’s test) was used for the calculation. For the analysis of time-dependent growth or decrease curves, a two-way ANOVA with a Bonferroni posttest was used. For Kaplan-Meier survival analysis, the log-rank test was used for comparative analysis. To analyze the correlation between the two variables, Pearson and Spearman correlation coefficient were used for the calculation. A value of *p* < 0.05 was indicated to determine the significant difference in each figure.

## Supplementary information

aj-checklist_CDDIS-22-2383-T

Supplementary information

Supplementary Table 1

Supplementary Table 2

Supplementary Table 3

Original Data File

## Data Availability

All datasets used in this study are publicly available. RNA sequencing data generated during the current study are available in the Gene Expression Omnibus (GEO) repository, under the accession number GSE193382.
