# Supplementary material for: CCN3/NOV promotes metastasis and tumor progression via GPNMB-induced EGFR activation in triple-negative breast cancer
Source: Cell Death Dis. 2023 Feb 3;14(2):81. doi: 10.1038/s41419-023-05608-3 (PMC9898537; doi:10.1038/s41419-023-05608-3)
Supplement: Supplementary file 2 — Supplementary information [file 41419_2023_5608_MOESM2_ESM.docx]

**Supplementary figures**

**Supplementary Figure 1.** **CCN3 highly expressed in TNBC and correlates with metastatic phenotype (continued from Fig.1).**


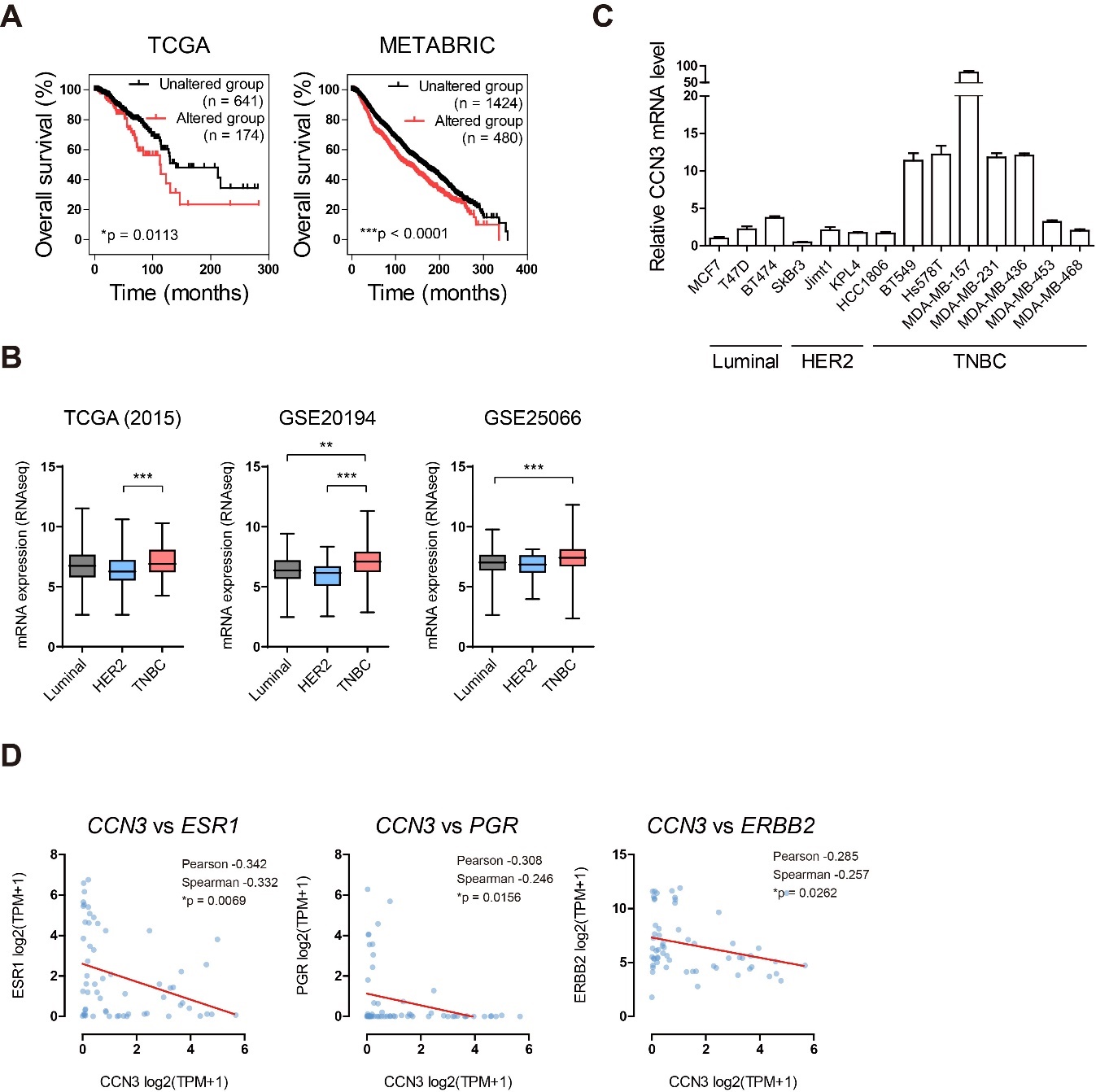


(A) Overall survival plot of *CCN3* altered/unaltered patients in the indicated dataset. P values were calculated with log-rank test. (B) *CCN3* mRNA expression of breast cancer patients. mRNA expression data were obtained from TCGA (luminal, n = 593; HER2, n = 120; TNBC, n = 82), GSE20194 (luminal, n = 161; HER2, n = 33; TNBC, n = 74) and GSE25066 (luminal, n = 291; HER2, n = 10; TNBC, n = 207) respectively. Expression values were clustered by breast cancer molecular subtype. P values were calculated with one-way ANOVA with a post-hoc Tukey’s multiple comparison test (**p < 0.005, ***p < 0.0005). (C) RT-qPCR analysis showed *CCN3* mRNA level of 14 breast cancer cell lines. Each value was normalized with GAPDH. Normalized mRNA expression of each cell line was compared with the value of MCF7. Mean ± SD (n = 3). (D) Correlation between *CCN3* mRNA expression and *ESR1/PGR/ERBB2* mRNA expression. Assessed data were from CCLE dataset.

**Supplementary Figure 2.** **CCN3 regulates cell migration, invasion and EMT phenotype (continued from Fig 2).**


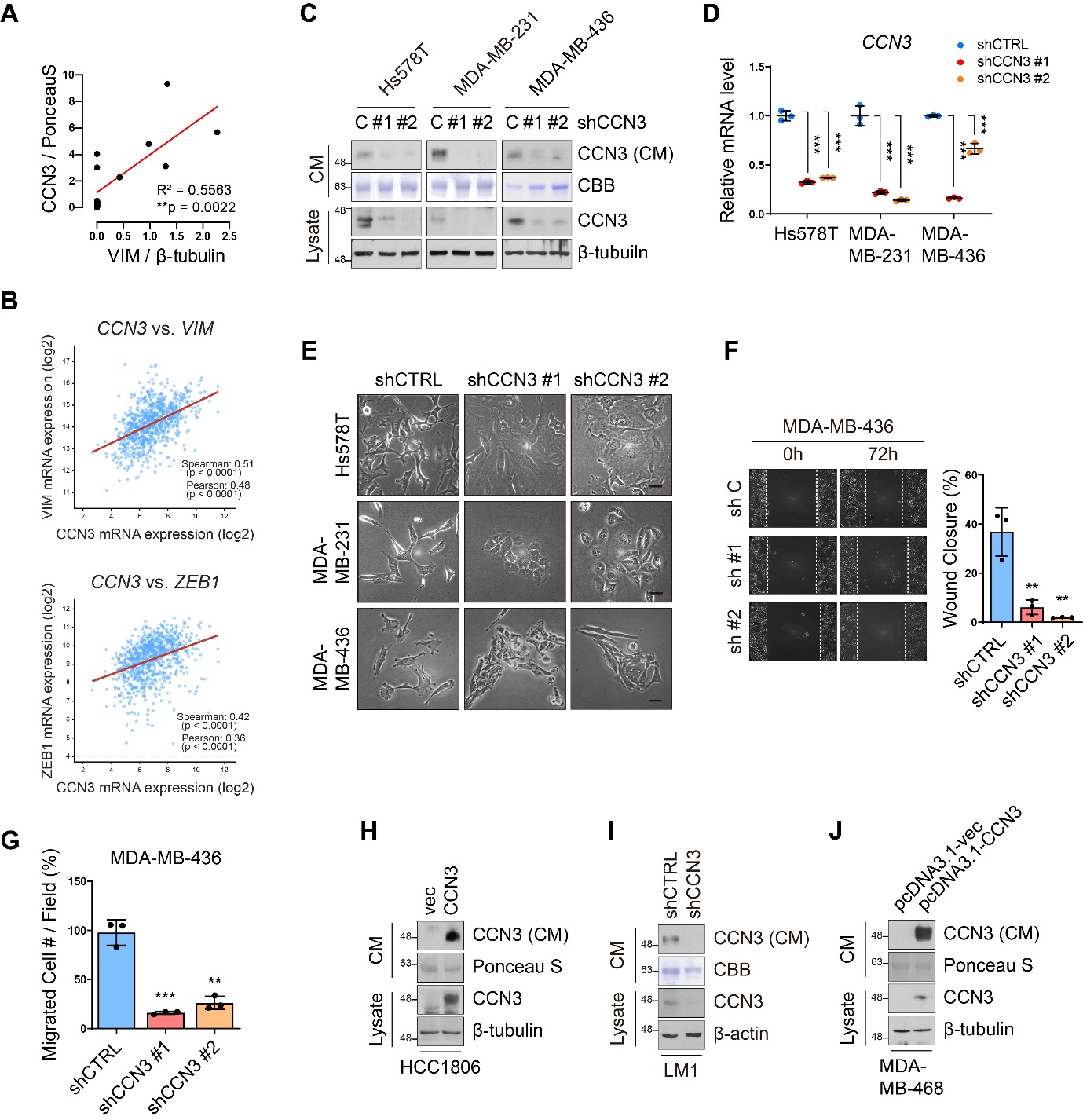


(A) Correlation between secreted CCN3 level and vimentin level in breast cancer cell lines. Secreted CCN3 level was normalized by ponceau S staining intensity and vimentin level was normalized by β-tubulin band intensity. (B) Correlation between *CCN3* mRNA expression and EMT marker (*ZEB1* and *VIM*) mRNA expression. Assessed data were from TCGA dataset (Cell, 2015) (C) Western blot analysis of whole cell lysate and precipitated protein from conditioned media with indicated antibody. β-tubulin was used as a loading control for whole cell lysate and Coomassie Brilliant Blue staining intensity was used as a loading control for conditioned media. CM; conditioned media. CBB; Coomassie brilliant blue. (D) RT-qPCR analysis showed CCN3 mRNA level. Each value was normalized with GAPDH. Mean ± SD (n = 3). P values were calculated with one-way ANOVA with a post-hoc Dunnett’s multiple comparisons test (***p < 0.0005). (E) Brightfield microscopic image indicated morphological change of CCN3 knockdown cell lines (scale bar = 50μm). (F) Wound-healing assay of MDA-MB-436 CCN3 knockdown cell lines. The width of the wound area was monitored with a microscope at 24 h intervals. Mean ± SD (n = 3). P values were calculated with two-way ANOVA with a Bonferroni posttest (*p < 0.05, **p < 0.005, ***p < 0.0005). (G) Transwell migration assays were assessed with MDA-MB-436 CCN3 knockdown cell lines (scale bar = 200μm). Each value was normalized with the value of control cell line. Mean ± SD (n = 3). P values were calculated with one-way ANOVA with a post-hoc Dunnett’s multiple comparisons test (***p < 0.0005). (H-J) Western blot analysis was performed to confirm the overexpression or knockdown of CCN3 expression in each cell line. β-tubulin and β-actin were used as a loading control for whole cell lysate and ponceau S staining and Coomassie Brilliant Blue staining intensity was used as a loading control for conditioned media. CM; conditioned media. CBB; Coomassie brilliant blue.

**Supplementary Figure 3. CCN3 promotes in vivo metastasis and proliferation of breast cancer cells.**

**
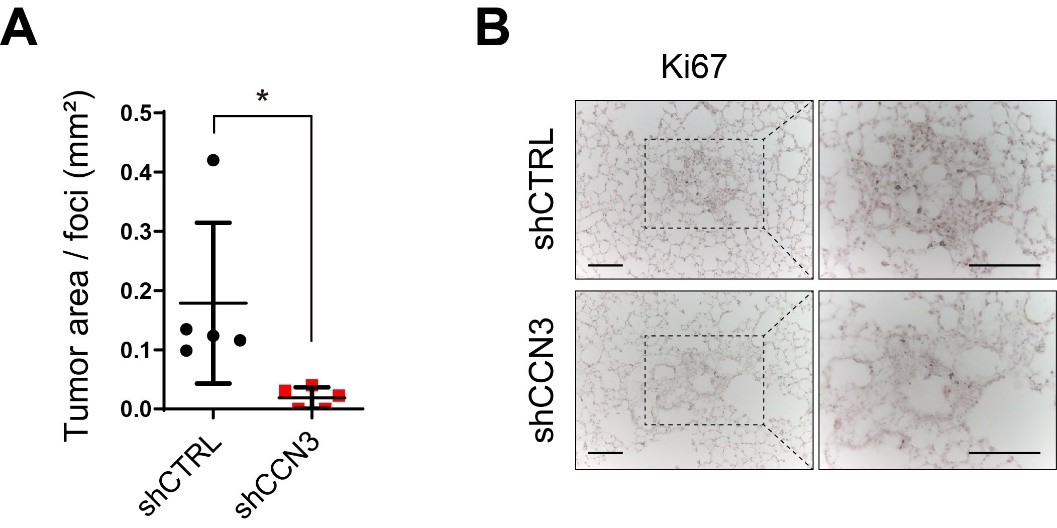
**

(A) Colonized tumor areas per each tumor foci on each lung section were calculated. Mean ± SD (n = 5). P value was calculated with two-tailed student t-test (***p < 0.0005). (B) Immunohistochemistry images of Ki67 expression in lung metastasis tissue (scale bar = 100μm). (C) CTC data 추가 예정

**Supplementary Figure 4. CCN3 enhances cancer stem cell-like phenotype (continued from Fig 3).**


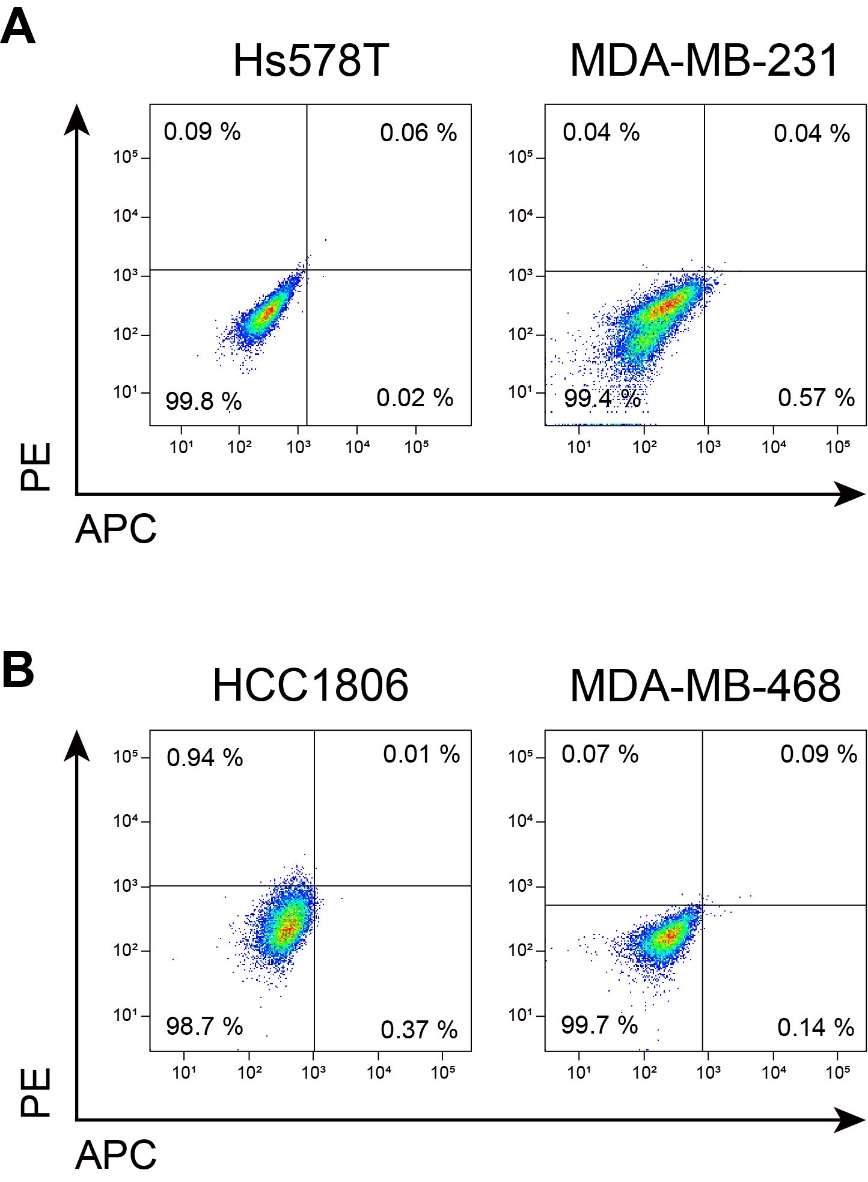


(A) FACS analysis of negative staining plot in the Hs578T and MDA-MB-231 CCN3 knockdown cell lines. (B) FACS analysis of negative staining plot in the HCC1806 and MDA-MB-468 CCN3 overexpression cell lines. Negative gating was adjusted for each positive population to be less than 1% of the total population.

**Supplementary Figure 5. Neutralization of CCN3 decreases cell viability in TNBC cells.**


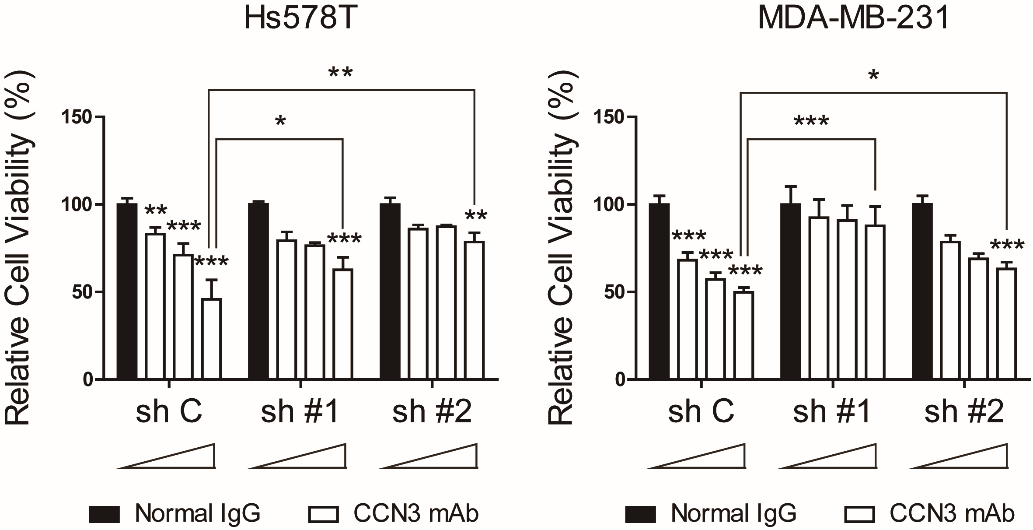


Hs578T and MDA-MB-231 control and CCN3 knockdown cell were treated with CCN3 monoclonal antibody. After 77h, cell viability was measured by MTT assay. Concentration of antibody was 5, 10, 20 ug/ml, respectively. Normal mouse IgG was used as negative control. Value of viability were normalized with the value of negative control. Mean ± SD (n = 4). P values of each concentration of antibody were calculated with Two-tailed student *t* test. P values of each cell lines were one-way ANOVA with a post-hoc Dunnett’s multiple comparisons test (*p < 0.05, **p < 0.005, ***p < 0.0005).

**Supplementary Figure 6. Immunohistochemical analysis of xenografted tumor.**


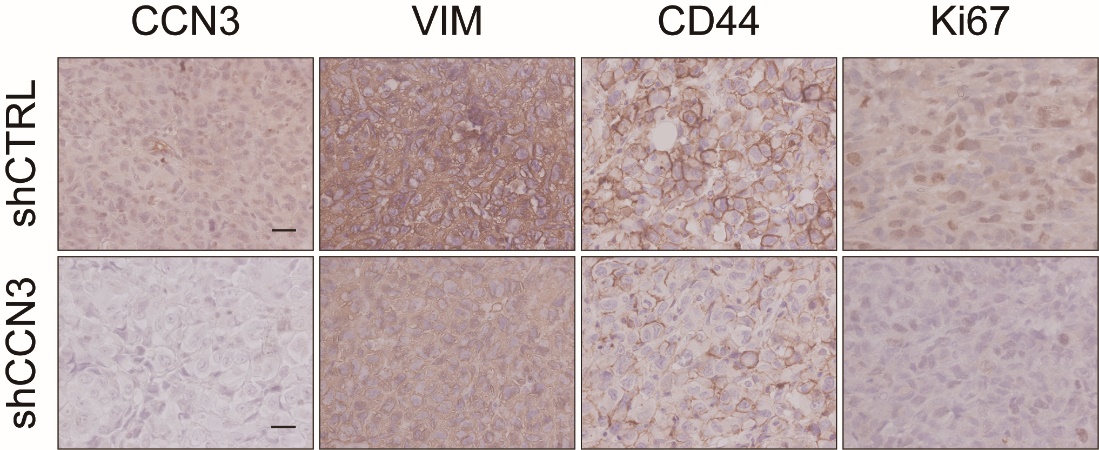


Immunohistochemistry images of CCN3, VIM, CD44 and Ki67 expression in xenograft tumor (scale bar = 20μm).

**Supplementary Figure 7. shRNA resistant CCN3 restores decreased proliferation of CCN3 knockdown cell lines.**


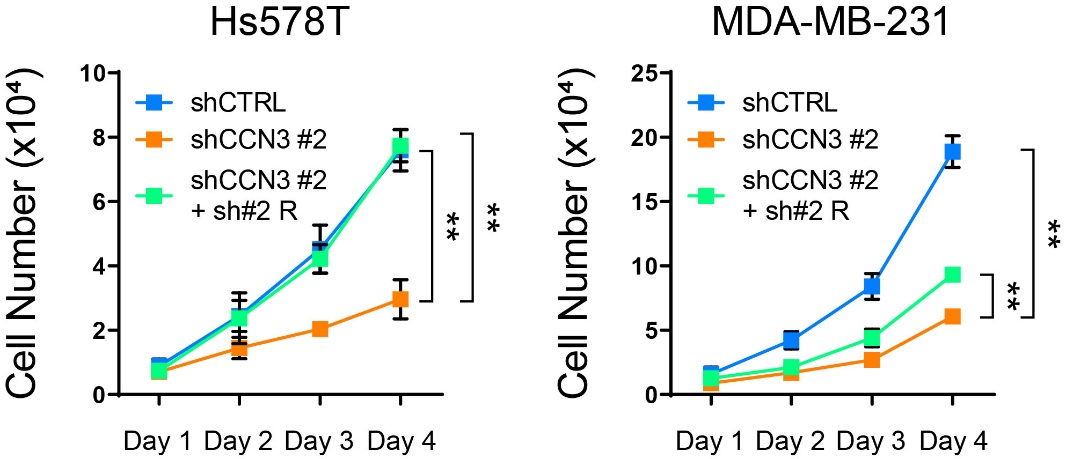


Cell proliferation assays were performed using Hs578T and MDA-MB-231 CCN3 knockdown cell lines after transfection with or without shRNA resistant CCN3. Mean ± SD (n = 3). P values were calculated with two-way ANOVA with a Bonferroni posttest (**p < 0.005, ***p < 0.0005).

**Supplementary Figure 8. EGFR expression positively correlates with CCN3 expression.**


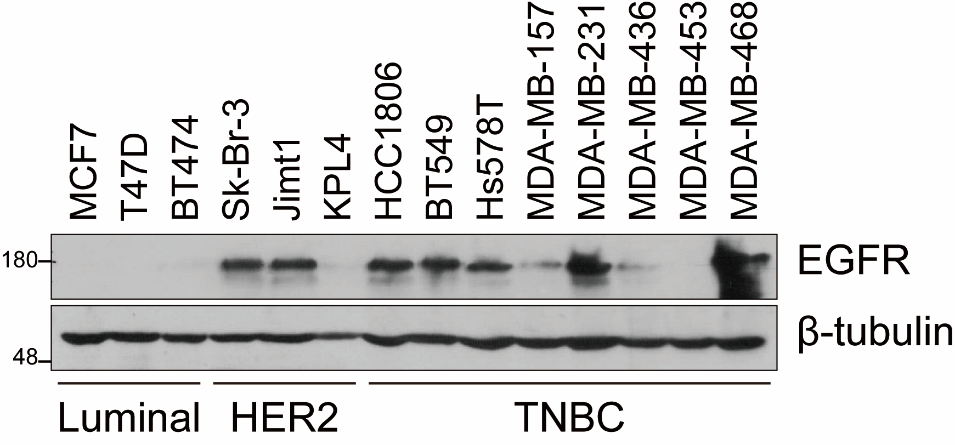


Western blot analysis of 14 breast cancer cell lines with indicated antibodies. β-tubulin was used as a loading control.

**Supplementary Figure 9. RNA-seq results indicated that CCN3 affects EGFR and stem cell-related gene expression.**


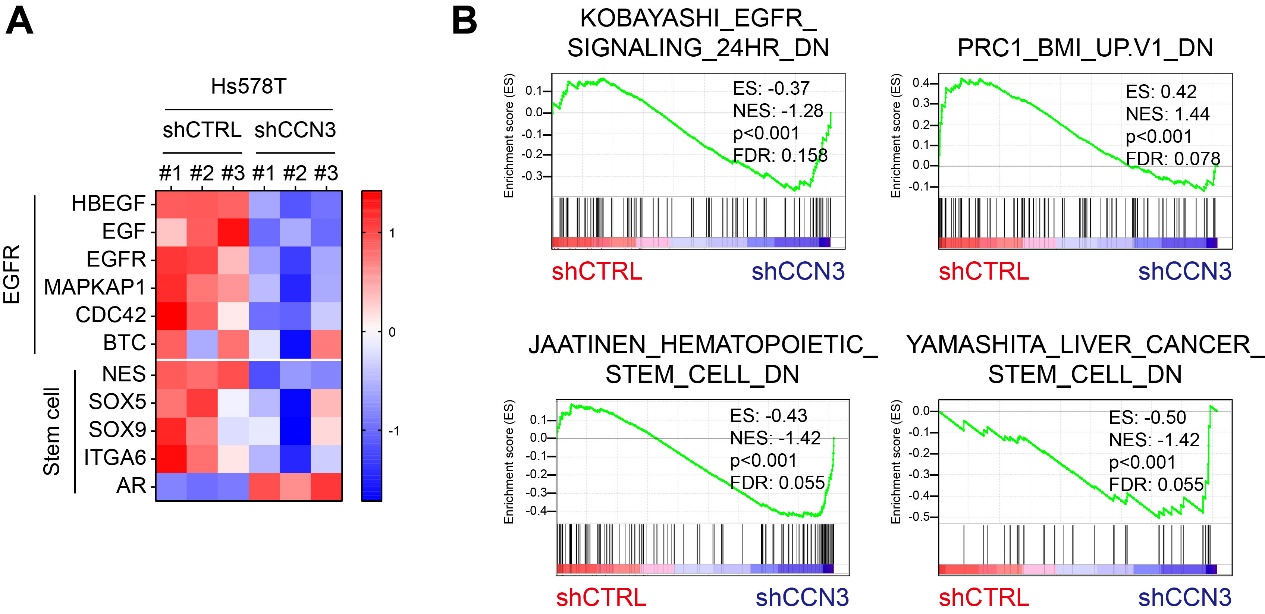


(A) The heatmap presents EGFR and stem cell-related genes altered by CCN3 in the RNA-seq data set. (B) GSEA was performed with mRNA expression data from RNA-seq dataset. Plots indicate that enrichment of EGFR and stem cell-related gene signatures were decreased in shCCN3 cell line. ES; enrichment score, NES; normalized enrichment score, FDR; false discovery rate.

**Supplementary Figure 10.** **Expression of GPNMB is positively correlated with expression of CCN3 and upregulated in TNBC patients.**


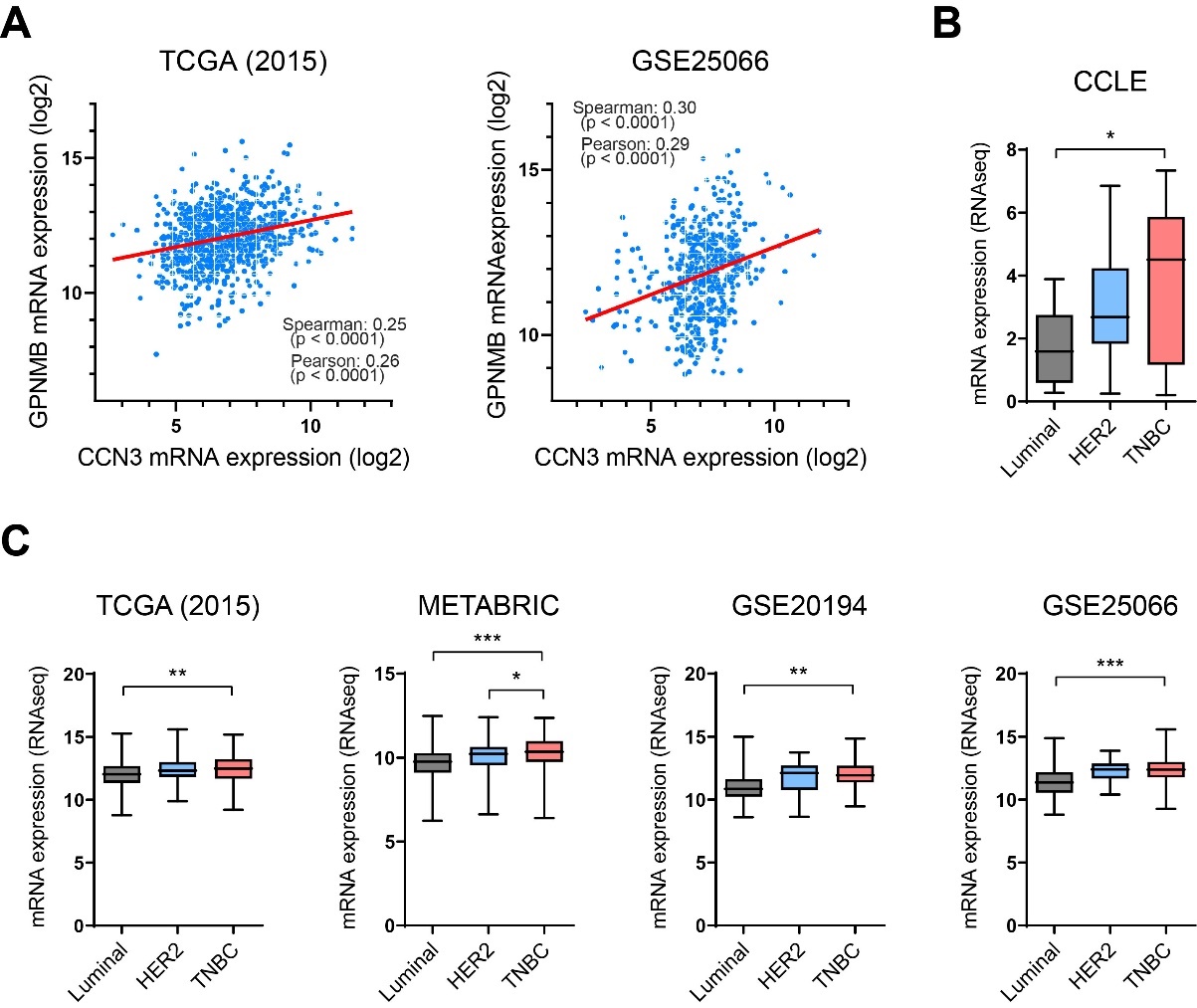


(A) Correlation between *CCN3* mRNA expression and *GPNMB* mRNA expression. Assessed data were from TCGA dataset (Cell, 2015) and GSE25066. (B) *GPNMB* mRNA expression of breast cancer cell lines. mRNA expression data were obtained from CCLE dataset. Expression values were clustered by breast cancer molecular subtype (luminal, n = 11; HER2, n = 19; TNBC, n = 33). P values were calculated with one-way ANOVA with a post-hoc Tukey’s multiple comparison test (*p < 0.05). (C) *GPNMB* mRNA expression of breast cancer patients. mRNA expression data were obtained from TCGA (luminal, n = 593; HER2, n = 120; TNBC, n = 82), METABRIC (luminal, n = 1355; HER2, n = 236; TNBC, n = 299), GSE20194 (luminal, n = 161; HER2, n = 33; TNBC, n = 74) and GSE25066 (luminal, n = 291; HER2, n = 10; TNBC, n = 207) respectively. Expression values were clustered by breast cancer molecular subtype. P values were calculated with one-way ANOVA with a post-hoc Tukey’s multiple comparison test (*p < 0.05, **p < 0.005, ***p < 0.0005).

**Supplementary Figure 11. Exogenous CCN3 promotes expression of GPNMB.**


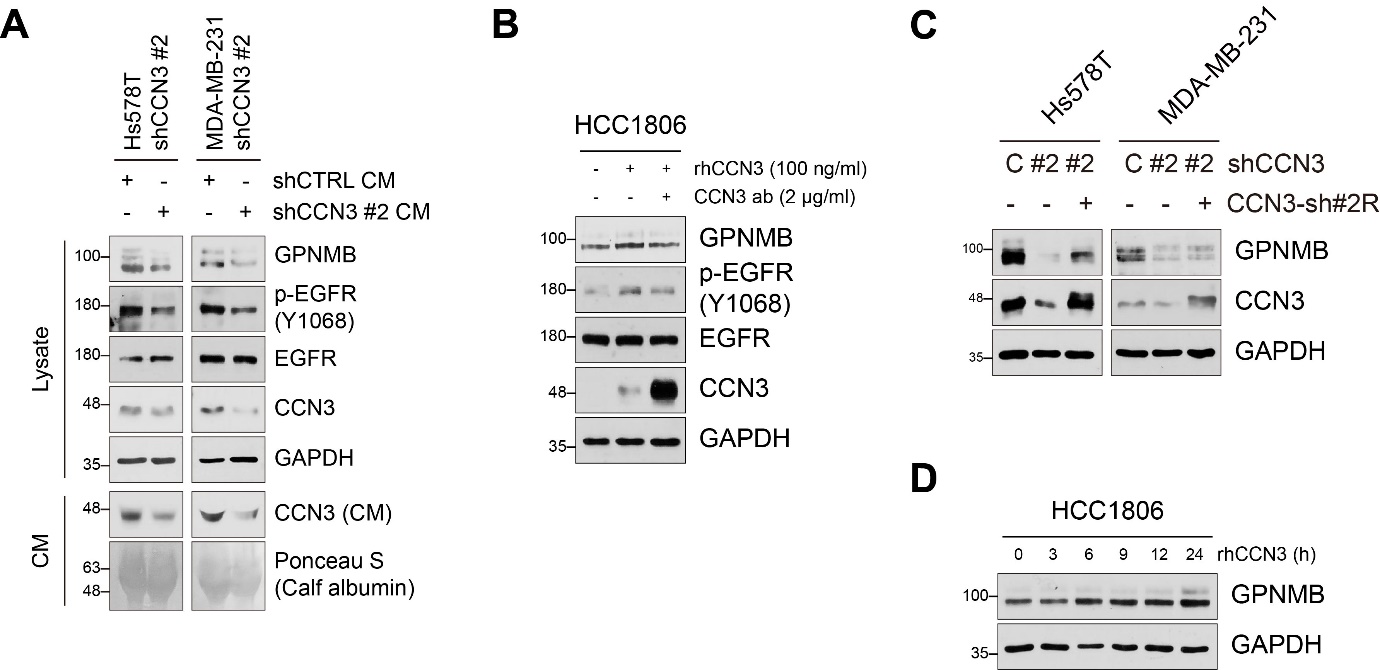


(A) Western blot analysis of conditioned media treated Hs578T and MDA-MB-231 CCN3 knockdown cell lines with indicated antibodies. GAPDH was used as a loading control for whole cell lysate and ponceau S staining intensity was used as a loading control for conditioned media. (B) Western blot analysis of rhCCN3 and CCN3 ab treated HCC1806 with indicated antibodies. GAPDH was used as a loading control (C) Western blot analysis of whole cell lysate with indicated antibody. CCN3 knockdown cell line restored their expression by transfection with shRNA resistant CCN3 construct. GAPDH was used as a loading control. (D) Western blot analysis of time-dependent treatment of rhCCN3 with HCC1806. rhCCN3 was treated at a concentration of 100 ng/ml. GAPDH was used as a loading control.

**Supplementary Figure 12. Knockdown of GPNMB drives anti-tumorigenic effect *in vitro* (continued from Fig 7)*.***


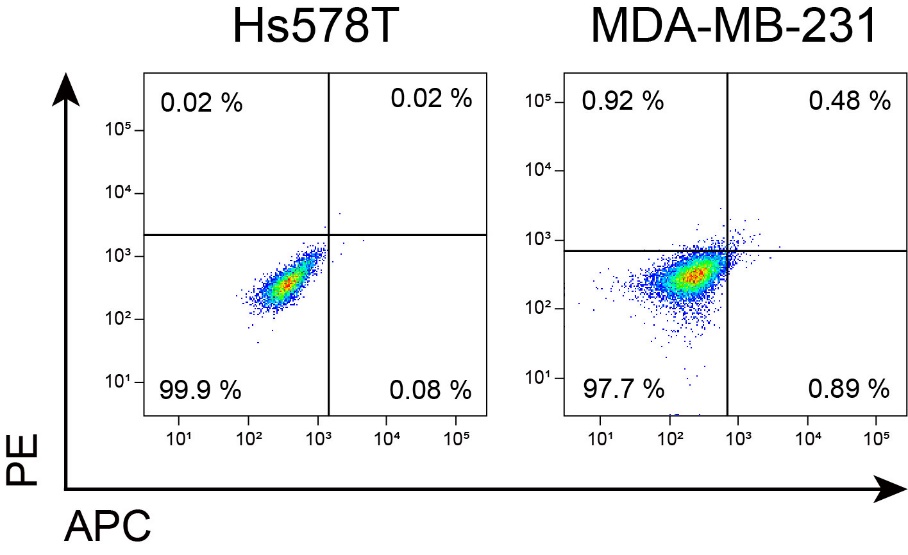


FACS analysis of negative staining plot in the Hs578T and MDA-MB-231 GPNMB knockdown cell lines. Negative gating was adjusted for each positive population to be less than 1% of the total population.

**Supplementary Figure 13. CCN3 knockdown downregulates MITF, but not TFEB and TFE3.**


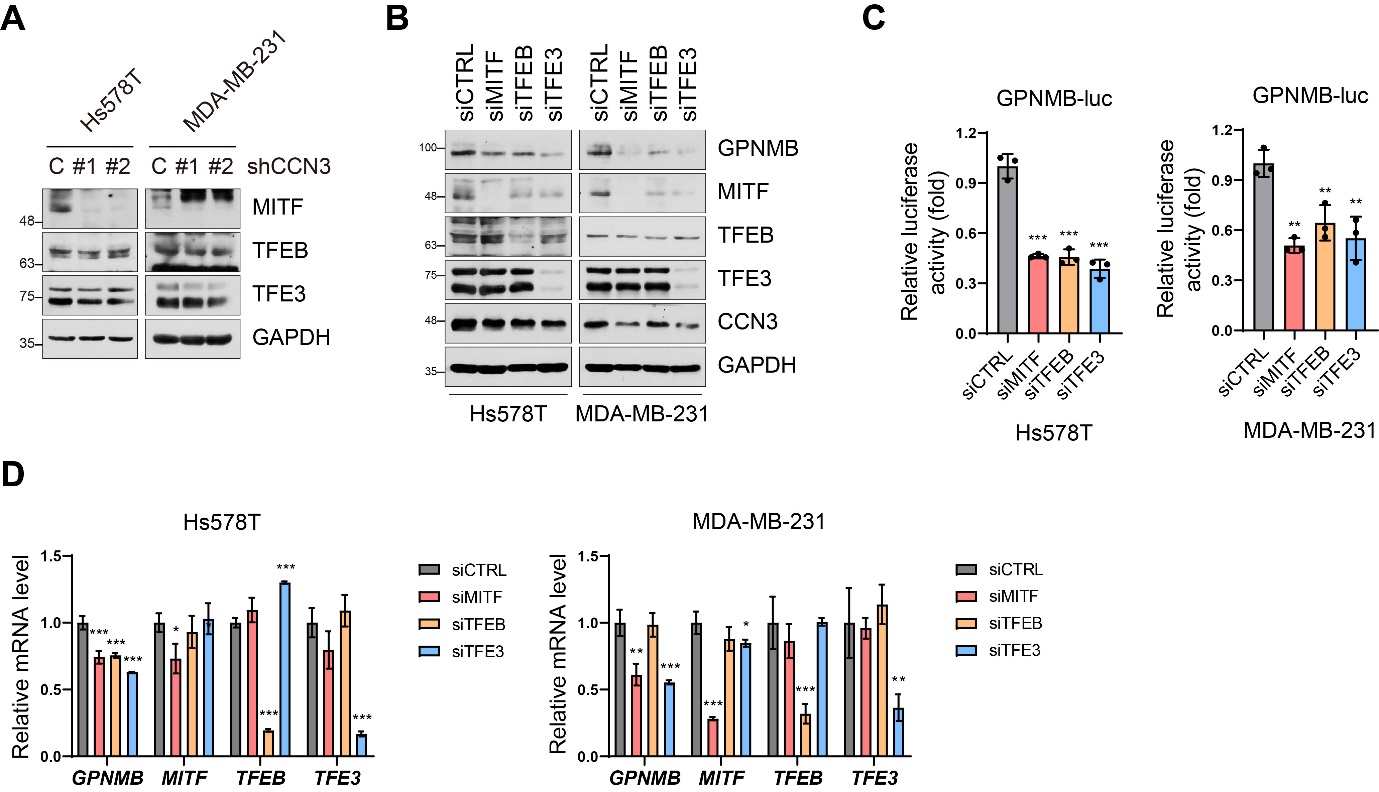


(A) Western blot analysis of whole cell lysate of CCN3 knockdown cell lines with indicated antibody. GAPDH was used as a loading control. (B) Western blot analysis of MITF, TFEB and TFE3 siRNA transfected Hs578T and MDA-MB-231. GAPDH was used as a loading control. (C) A dual luciferase assay was performed using pGL3-GPNMB-luc construct. pCMV-RL was used as an internal control. Mean ± SD (n = 3). P values were calculated with one-way ANOVA with a post-hoc Dunnett’s multiple comparison test (**p < 0.005, ***p < 0.0005). (D) RT-qPCR analysis showed mRNA level of indicated genes in MITF, TFEB and TFE3 siRNA transfected Hs578T and MDA-MB-231. Each value was normalized with GAPDH. Mean ± SD (n = 3). P values were calculated with one-way ANOVA with a post-hoc Dunnett’s multiple comparison test (*p < 0.05, **p < 0.005, ***p < 0.0005).

**Supplementary Figure 14. knockdown of β-catenin decreases GPNMB expression and following signaling pathway.**


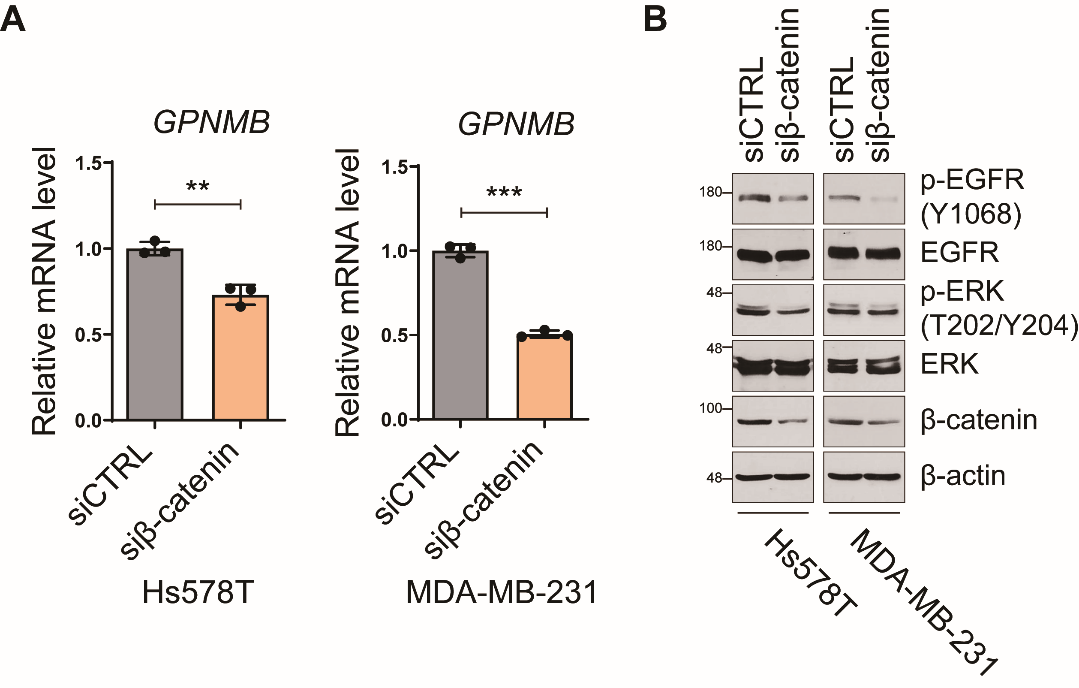


(A) RT-qPCR analysis showed GPNMB mRNA level in β-catenin siRNA transfected Hs578T and MDA-MB-231. Each value was normalized with GAPDH. Mean ± SD (n = 3). P values were calculated with Two-tailed student *t* test. (**p < 0.005, ***p < 0.0005). (B) Western blot analysis of β-catenin siRNA transfected Hs578T and MDA-MB-231 with indicated antibodies. β-actin was used as a loading control.

**Supplementary Figure 15. LiCl increases GPNMB expression and MITF protein level.**


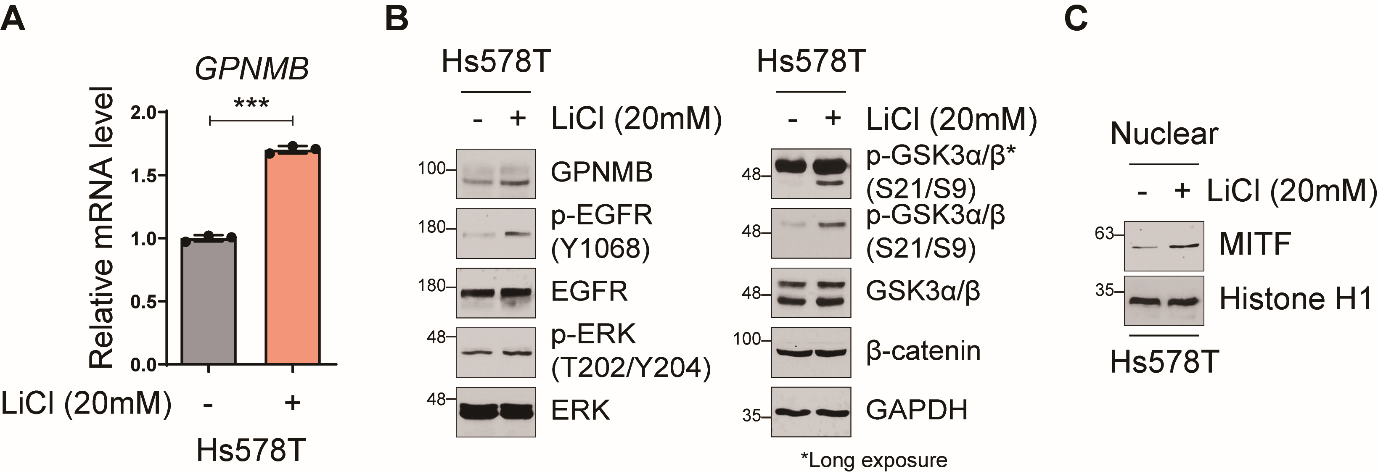


(A) RT-qPCR analysis showed GPNMB mRNA level in LiCl treated Hs578T. Each value was normalized with GAPDH. Mean ± SD (n = 3). P values were calculated with Two-tailed student *t* test. (**p < 0.005, ***p < 0.0005). (B) Western blot analysis of LiCl treated Hs578T with indicated antibodies. GAPDH was used as a loading control. (C) Western blot analysis of lysate isolated into cytoplasm and nuclear of LiCl treated Hs578T using the indicated antibodies. β-actin and histone H1 were used as a loading control for cytosol and nuclear, respectively.

**Supplementary Figure 16. Correlation between CCN3 and Wnt signaling.**


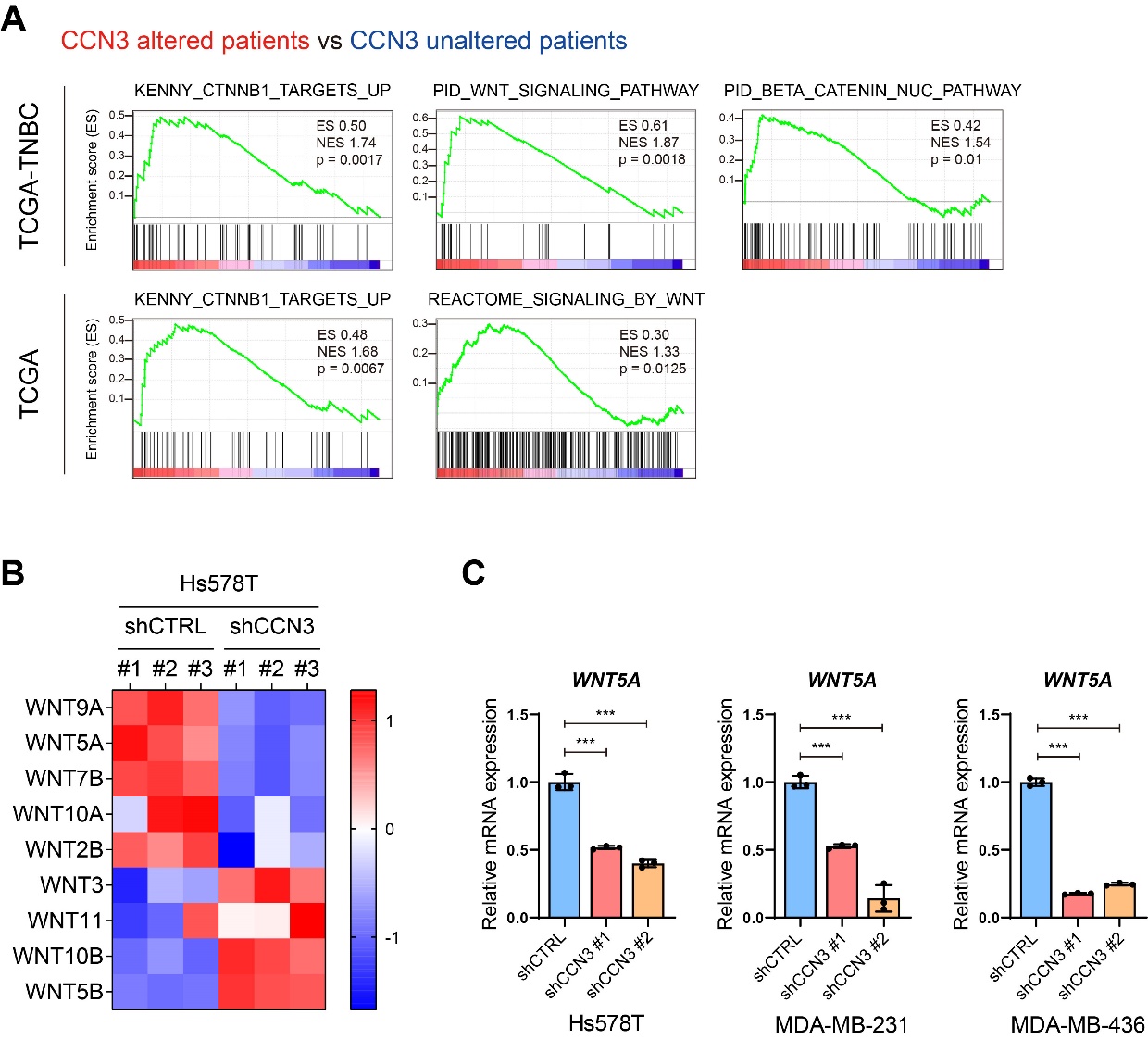


(A) GSEA was performed with mRNA expression data from TCGA (Cell, 2015) dataset. ES; enrichment score, NES; normalized enrichment score. (B) The heatmap presents Wnt ligand expression altered by CCN3 in the RNA-seq data set. (C) RT-qPCR analysis showed the *WNT5A* mRNA level in Hs578T, MDA-MB-231 and MDA-MB-436 CCN3 knockdown cell lines. Each value was normalized with GAPDH. Mean ± SD (n = 3). P values were calculated with one-way ANOVA with a post-hoc Dunnett’s multiple comparison test (***p < 0.0005).

**Supplementary tables**

Supplementary Table.1 List of antibodies

Supplementary Table.2 List of shRNA sequences

Supplementary Table.3 List of primers for plasmid construction
