# Supplementary material for: CCN3/NOV promotes metastasis and tumor progression via GPNMB-induced EGFR activation in triple-negative breast cancer
Source: Cell Death Dis. 2023 Feb 3;14(2):81. doi: 10.1038/s41419-023-05608-3 (PMC9898537; doi:10.1038/s41419-023-05608-3)

Figure.1

Fig1F CCN3 (CM)

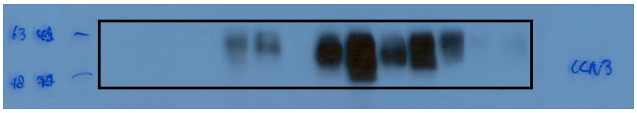

Fig1F Ponceau S

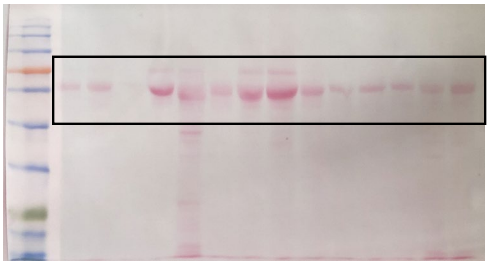

Fig1F E-cad

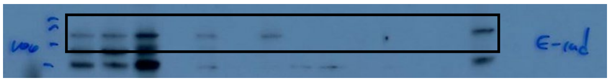

Fig1F VIM

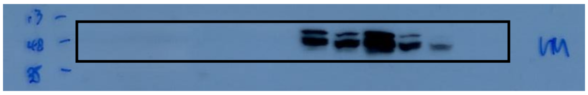

Fig1F CCN3

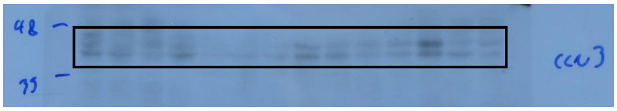

Fig1F β-tubulin

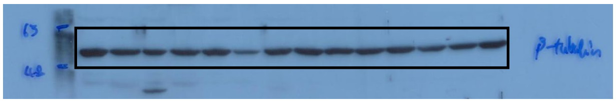

Fig1F CCN3 (RT-PCR)

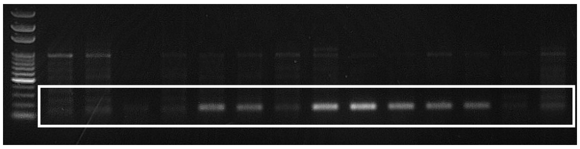

Fig1F GAPDH (RT-PCR)

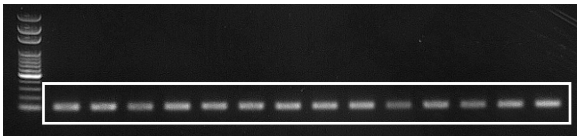

Figure.2

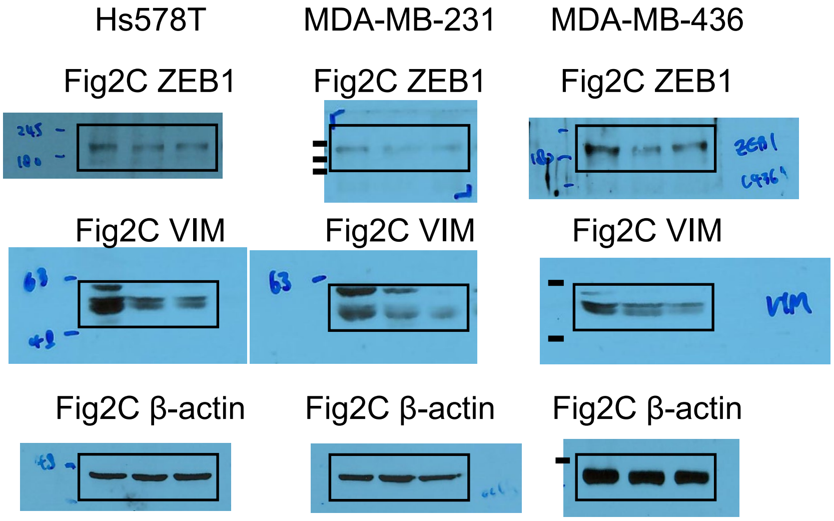
Hs578T
MDA-MB-231
MDA-MB-436
Fig2C ZEB1
Fig2C ZEB1
Fig2C ZEB1
Fig2C VIM
Fig2C VIM
Fig2C VIM
Fig2C β-actin
Fig2C β-actin
Fig2C β-actin

Figure.3

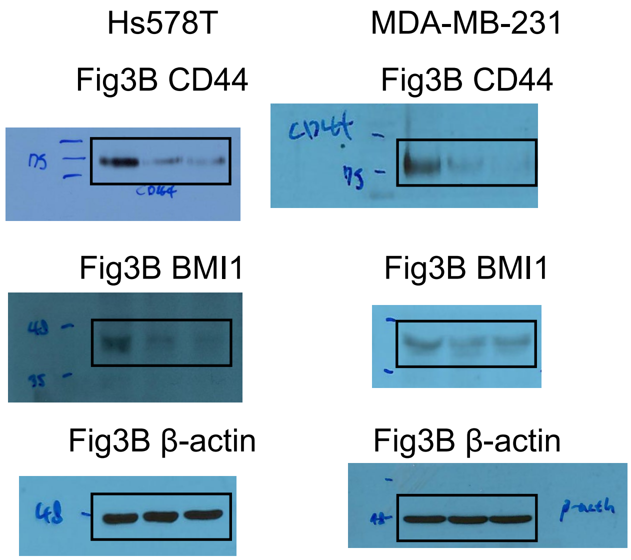
Hs578T
MDA-MB-231
Fig3B CD44
Fig3B CD44
Fig3B BMI1
Fig3B BMI1
Fig3B β-actin
Fig3B β-actin

Figure.5

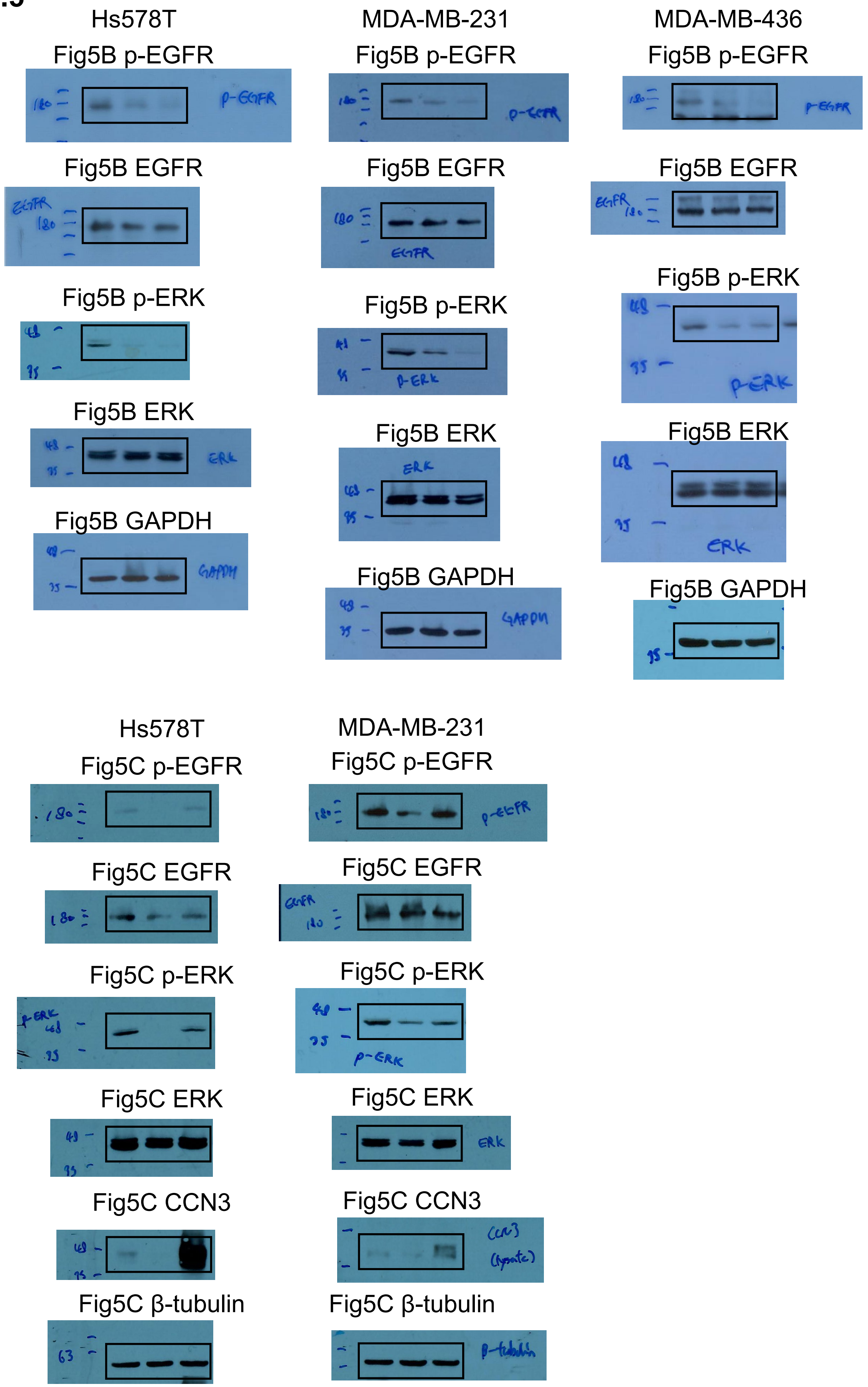
Hs578T
MDA-MB-231
MDA-MB-436
Fig5B p-EGFR
Fig5B p-EGFR
Fig5B p-EGFR
Fig5B EGFR
Fig5B EGFR
Fig5B EGFR
Fig5B p-ERK
Fig5B p-ERK
Fig5B p-ERK
Fig5B ERK
Fig5B ERK
Fig5B ERK
Fig5B GAPDH
Fig5B GAPDH
Fig5B GAPDH
Hs578T
MDA-MB-231
Fig5C p-EGFR
Fig5C p-EGFR
Fig5C EGFR
Fig5C EGFR
Fig5C p-ERK
Fig5C p-ERK
Fig5C ERK
Fig5C ERK
Fig5C CCN3
Fig5C CCN3
Fig5C β-tubulin
Fig5C β-tubulin

Figure.6

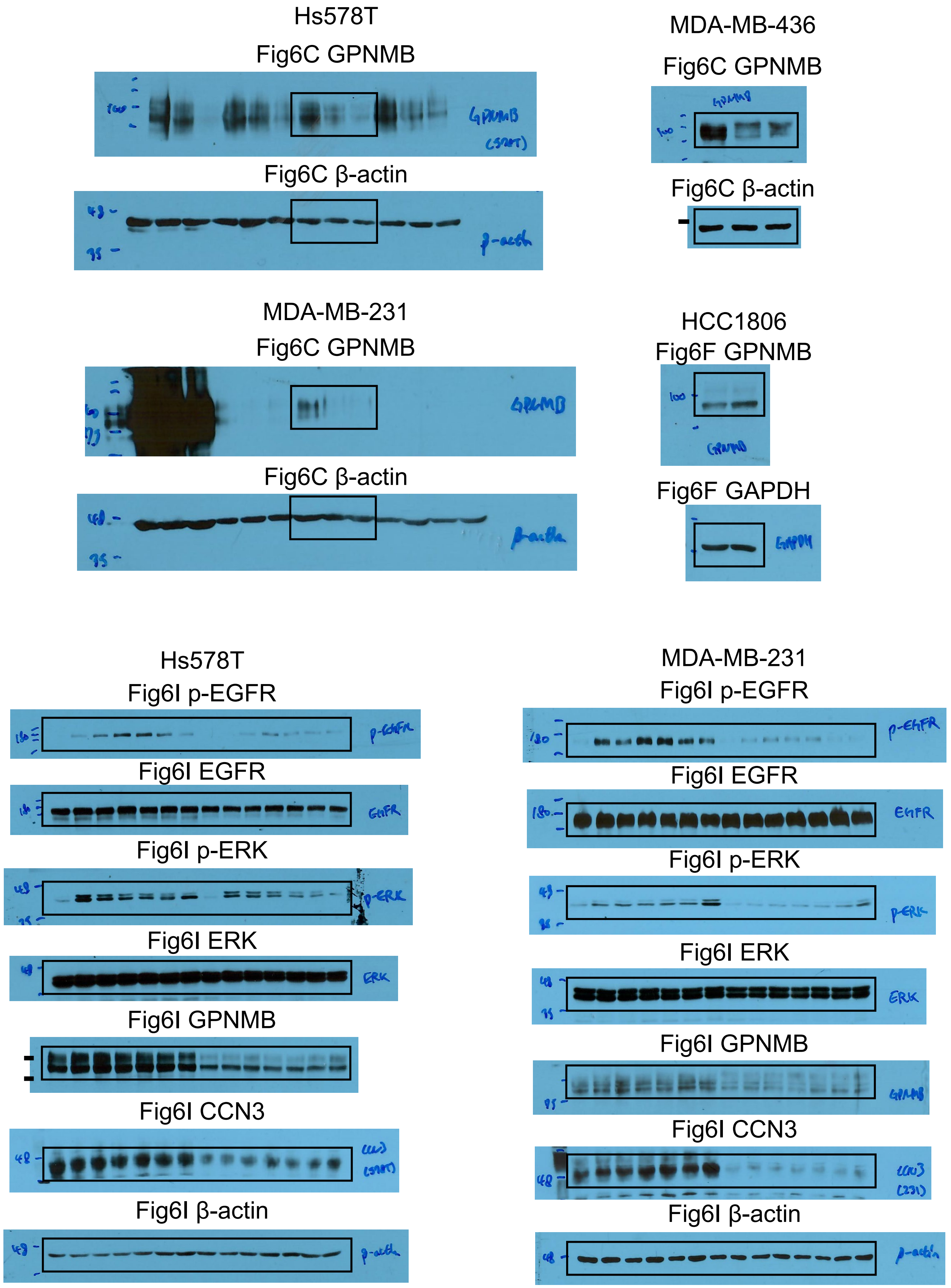

Hs578T
Fig6C GPNMB
Fig6C β-actin
MDA-MB-436
Fig6C GPNMB
Fig6C β-actin
MDA-MB-231
Fig6C GPNMB
Fig6C β-actin
HCC1806
Fig6F GPNMB
Fig6F GAPDH
Hs578T
Fig6I p-EGFR
Fig6I EGFR
Fig6I p-ERK
Fig6I ERK
Fig6I GPNMB
Fig6I CCN3
Fig6I β-actin
MDA-MB-231
Fig6I p-EGFR
Fig6I EGFR
Fig6I p-ERK
Fig6I ERK
Fig6I GPNMB
Fig6I CCN3
Fig6I β-actin

Figure.7

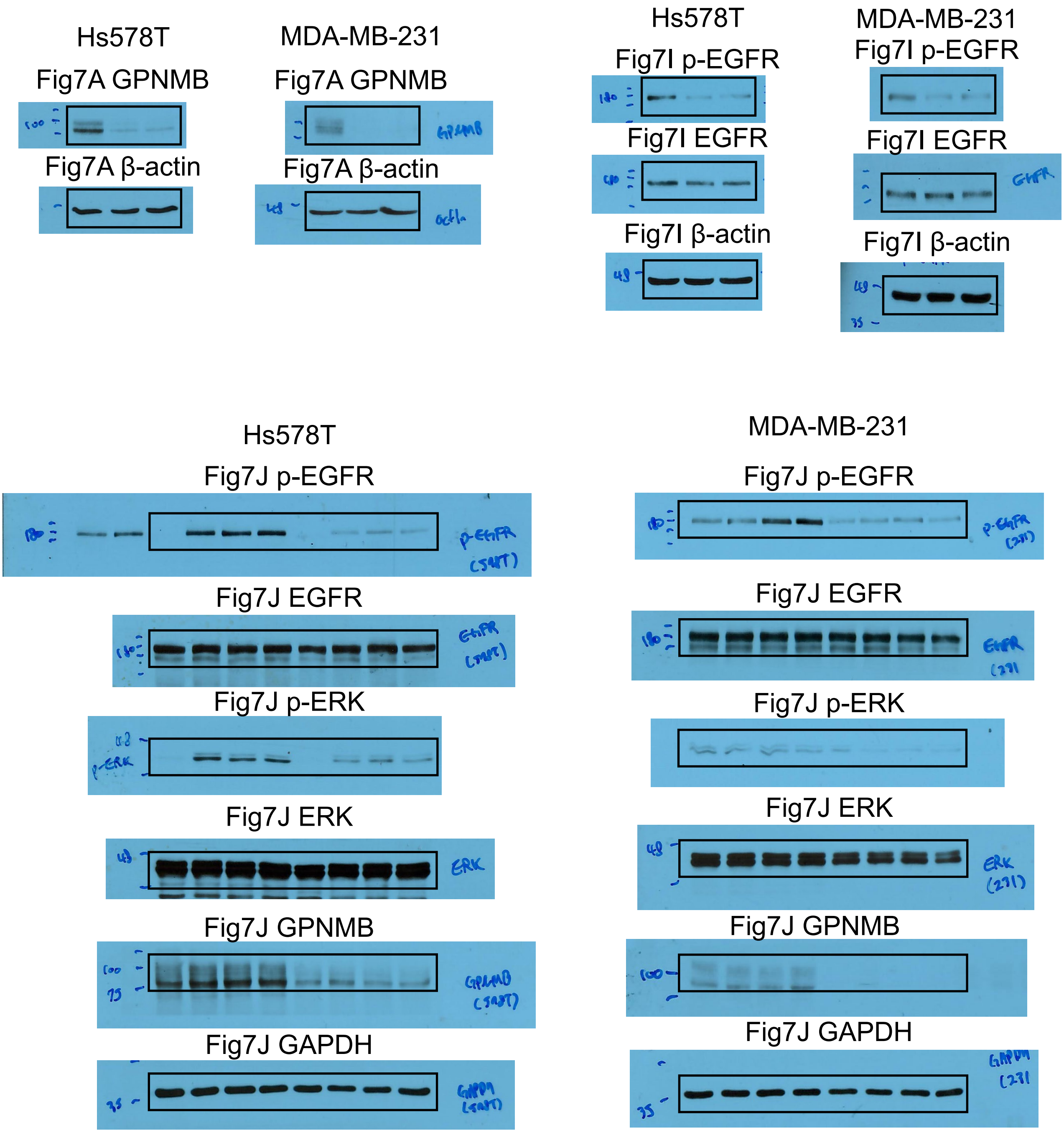
Hs578T
Fig7A GPNMB
Fig7A β-actin
MDA-MB-231
Fig7A GPNMB
Fig7A β-actin
Hs578T
Fig7I p-EGFR
Fig7I EGFR
Fig7I β-actin
MDA-MB-231
Fig7I p-EGFR
Fig7I EGFR
Fig7I β-actin
Hs578T
Fig7J p-EGFR
Fig7J EGFR
Fig7J p-ERK
Fig7J ERK
Fig7J GPNMB
Fig7J GAPDH
MDA-MB-231
Fig7J p-EGFR
Fig7J EGFR
Fig7J p-ERK
Fig7J ERK
Fig7J GPNMB
Fig7J GAPDH

Figure.8

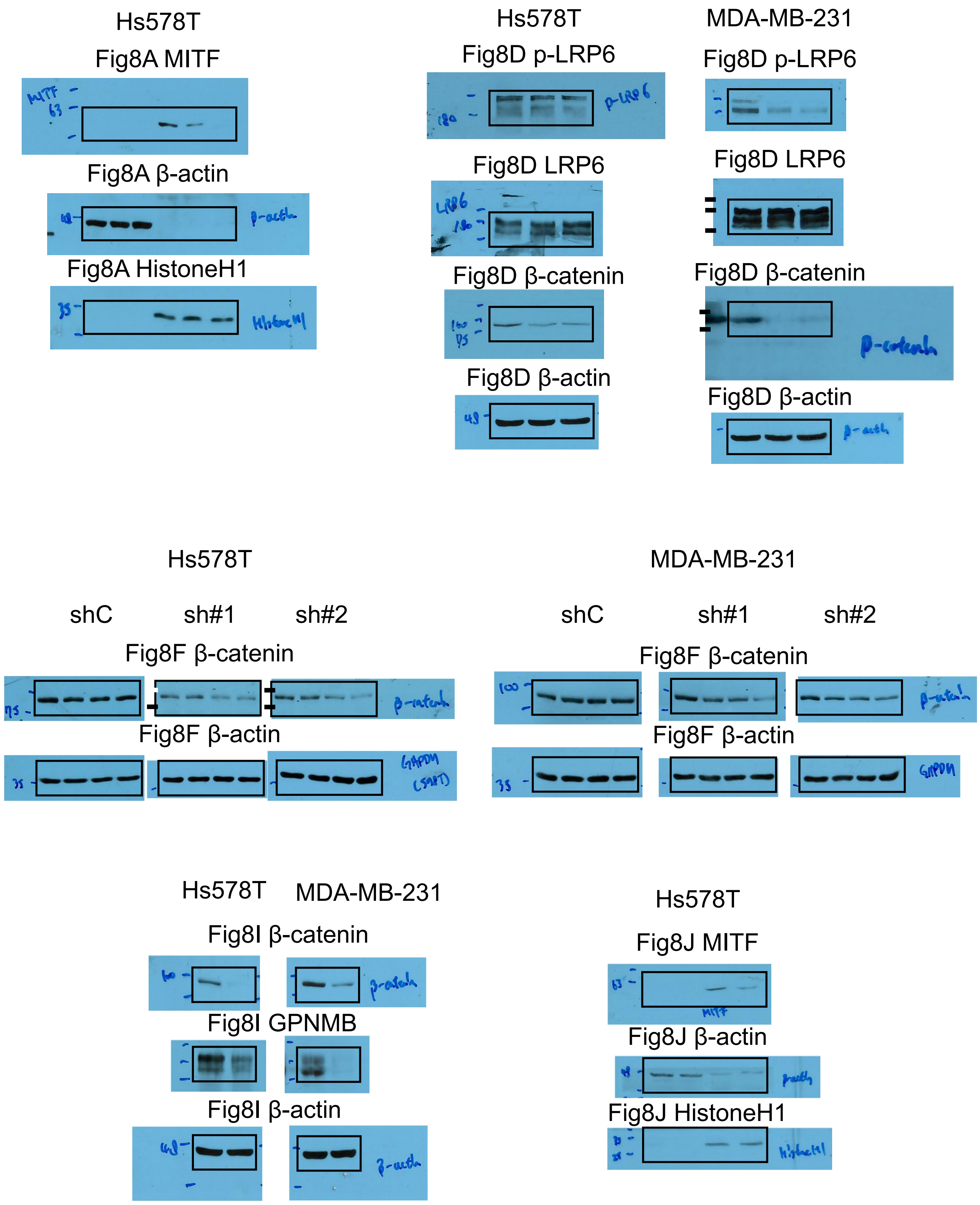
Hs578T
Fig8A MITF
Fig8A β-actin
Fig8A HistoneH1
Hs578T
Fig8D p-LRP6
Fig8D LRP6
Fig8D β-catenin
Fig8D β-actin
MDA-MB-231
Fig8D p-LRP6
Fig8D LRP6
Fig8D β-catenin
Fig8D β-actin
Hs578T
shC
sh#1
sh#2
Fig8F β-catenin
Fig8F β-actin
MDA-MB-231
shC
sh#1
sh#2
Fig8F β-catenin
Fig8F β-actin
Hs578T
MDA-MB-231
Fig8I β-catenin
Fig8I GPNMB
Fig8I β-actin
Hs578T
Fig8J MITF
Fig8J β-actin
Fig8J HistoneH1

Figure S2

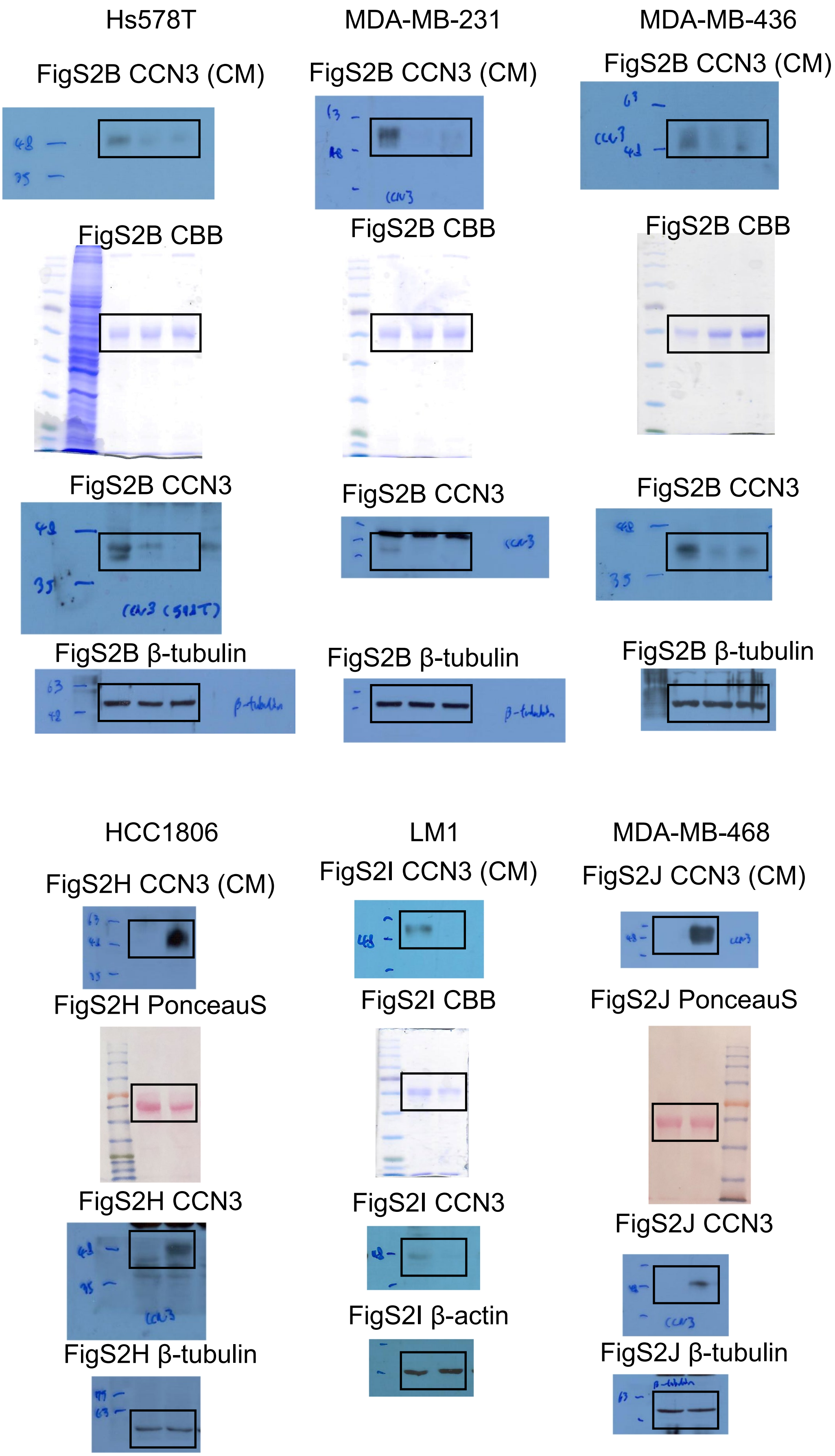
Hs578T
MDA-MB-231
MDA-MB-436
FigS2B CCN3 (CM)
FigS2B CCN3 (CM)
FigS2B CCN3 (CM)
FigS2B CBB
FigS2B CBB
FigS2B CBB
FigS2B CCN3
FigS2B CCN3
FigS2B CCN3
FigS2B β-tubulin
FigS2B β-tubulin
FigS2B β-tubulin
HCC1806
LM1
MDA-MB-468
FigS2H CCN3 (CM)
FigS2I CCN3 (CM)
FigS2J CCN3 (CM)
FigS2H PonceauS
FigS2I CBB
FigS2J PonceauS
FigS2H CCN3
FigS2I CCN3
FigS2J CCN3
FigS2I β-actin
FigS2H β-tubulin
FigS2J β-tubulin

Figure S8

FigS5 EGFR

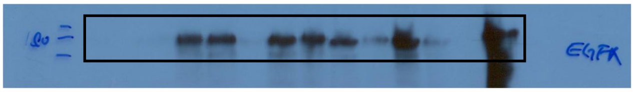

FigS5 β-tubulin

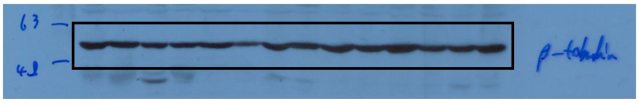

Figure S11

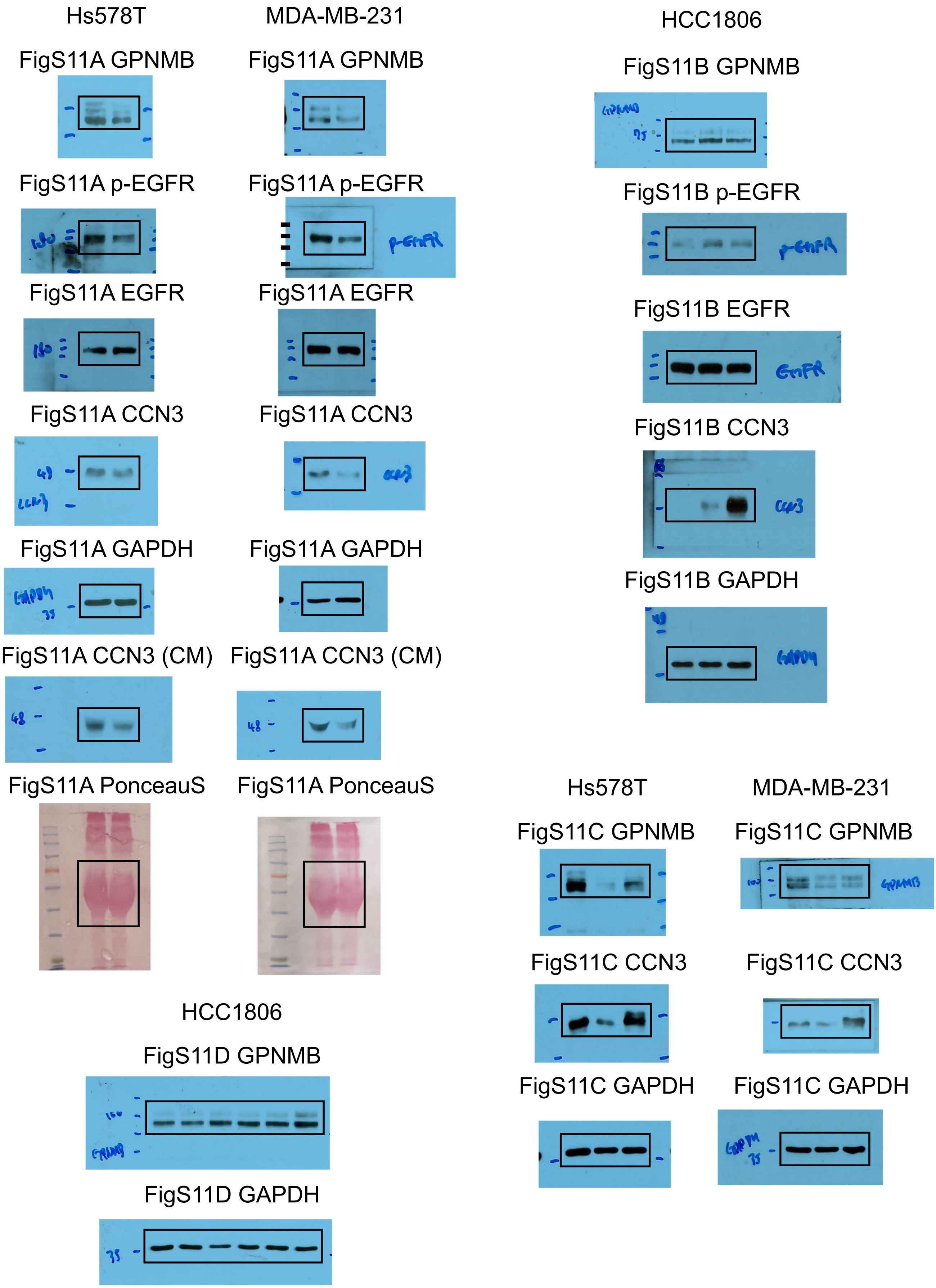
Hs578T
MDA-MB-231
HCC1806
FigS11A GPNMB
FigS11A GPNMB
FigS11B GPNMB
FigS11A p-EGFR
FigS11A p-EGFR
FigS11B p-EGFR
FigS11A EGFR
FigS11A EGFR
FigS11B EGFR
FigS11A CCN3
FigS11A CCN3
FigS11B CCN3
FigS11A GAPDH
FigS11A GAPDH
FigS11B GAPDH
FigS11A CCN3 (CM)
FigS11A CCN3 (CM)
FigS11A PonceauS
FigS11A PonceauS
Hs578T
MDA-MB-231
FigS11C GPNMB
FigS11C GPNMB
FigS11C CCN3
FigS11C CCN3
FigS11C GAPDH
FigS11C GAPDH
HCC1806
FigS11D GPNMB
FigS11D GAPDH

Figure S13

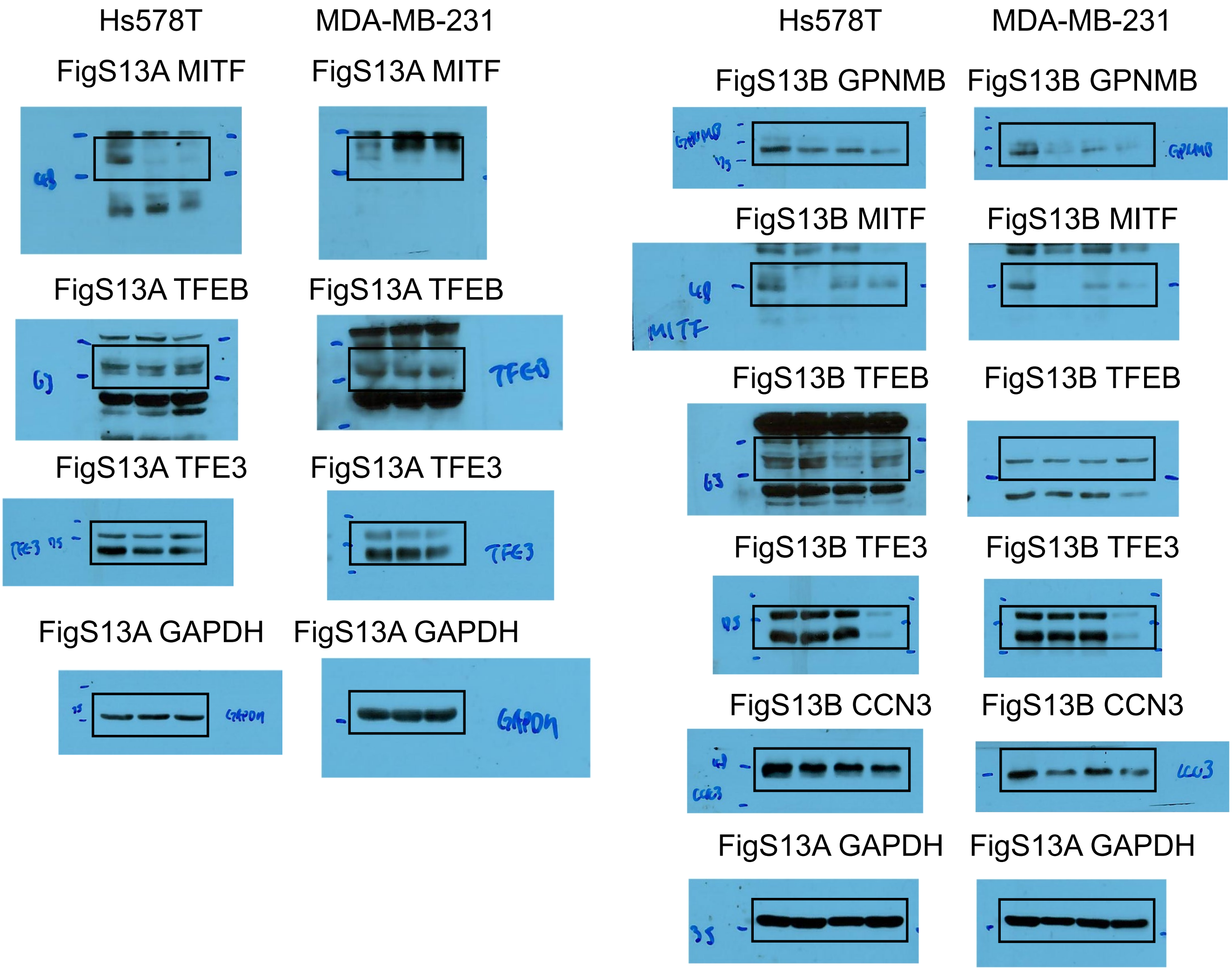

Hs578T
MDA-MB-231
FigS13A MITF
FigS13A MITF
FigS13A TFEB
FigS13A TFEB
FigS13A TFE3
FigS13A TFE3
FigS13A GAPDH
FigS13A GAPDH
Hs578T
MDA-MB-231
FigS13B GPNMB
FigS13B GPNMB
FigS13B MITF
FigS13B MITF
FigS13B TFEB
FigS13B TFEB
FigS13B TFE3
FigS13B TFE3
FigS13B CCN3
FigS13B CCN3
FigS13A GAPDH
FigS13A GAPDH

Figure S14

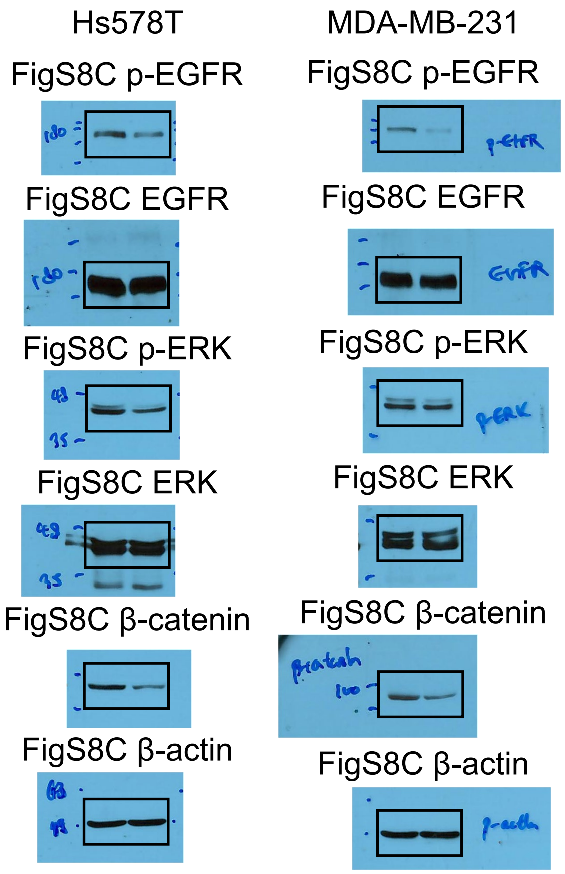
Hs578T
MDA-MB-231
FigS8C p-EGFR
FigS8C p-EGFR
FigS8C EGFR
FigS8C EGFR
FigS8C p-ERK
FigS8C p-ERK
FigS8C ERK
FigS8C ERK
FigS8C β-catenin
FigS8C β-catenin
FigS8C β-actin
FigS8C β-actin

Figure S15

Hs578T

FigS8E GPNMB

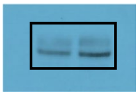

FigS8E p-EGFR

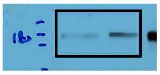

FigS8E EGFR

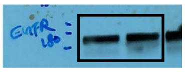

FigS8E p-ERK

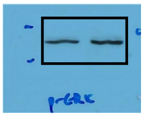

FigS8E ERK

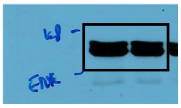

FigS8E p-GSK3α/β (long exposure)

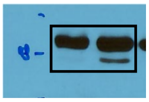

FigS8E p-GSK3α/β

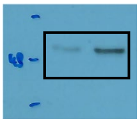

FigS8E GSK3α/β

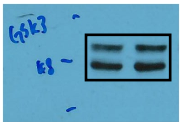

FigS8E β-catenin

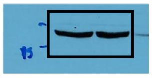

FigS8E GAPDH

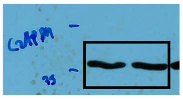

Hs578T

FigS8F MITF

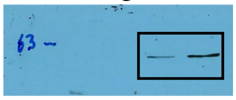

FigS8F HistoneH1

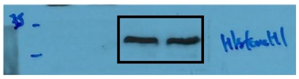

Supplement: Supplementary file 6 — Original Data File [file 41419_2023_5608_MOESM6_ESM.pdf]
